# Impact of Intestinal Microbiota on Growth and Feed Efficiency in Pigs: A Review

**DOI:** 10.3390/microorganisms8121886

**Published:** 2020-11-28

**Authors:** Gillian E. Gardiner, Barbara U. Metzler-Zebeli, Peadar G. Lawlor

**Affiliations:** 1 Department of Science, Waterford Institute of Technology, X91 K0EK Co. Waterford, Ireland; 2 Unit Nutritional Physiology, Institute of Physiology, Pathophysiology and Biophysics, Department of Biomedical Sciences, University of Veterinary Medicine Vienna, 1210 Vienna, Austria; barbara.metzler@vetmeduni.ac.at; 3 Teagasc, Pig Development Department, Animal and Grassland Research and Innovation Centre, Moorepark, Fermoy, P61 C996 Co. Cork, Ireland; peadar.lawlor@teagasc.ie

**Keywords:** gut, microbiome, swine, intestine, productivity, trait, bacterial taxa, microbial metabolite signalling, mucosal immune response

## Abstract

This review summarises the evidence for a link between the porcine intestinal microbiota and growth and feed efficiency (FE), and suggests microbiota-targeted strategies to improve productivity. However, there are challenges in identifying reliable microbial predictors of host phenotype; environmental factors impact the microbe–host interplay, sequential differences along the intestine result in segment-specific FE- and growth-associated taxa/functionality, and it is often difficult to distinguish cause and effect. However, bacterial taxa involved in nutrient processing and energy harvest, and those with anti-inflammatory effects, are consistently linked with improved productivity. In particular, evidence is emerging for an association of *Treponema* and methanogens such as *Methanobrevibacter* in the small and large intestines and *Lactobacillus* in the large intestine with a leaner phenotype and/or improved FE. Bacterial carbohydrate and/or lipid metabolism pathways are also generally enriched in the large intestine of leaner pigs and/or those with better growth/FE. Possible microbial signalling routes linked to superior growth and FE include increased intestinal propionate production and reduced inflammatory response. In summary, the bacterial taxa and/or metabolic pathways identified here could be used as biomarkers for FE/growth in pigs, the taxa exploited as probiotics or the taxa/functionality manipulated via dietary/breeding strategies in order to improve productivity in pigs.

## 1. Introduction

The pig intestinal microbiota is undeniably of critical importance to its host, conferring numerous services, such as nutrient digestion, disease resistance and production of vitamins and beneficial metabolites [1]. Therefore, it is not unreasonable to suggest that the pig gut microbiome is likely to impact growth and feed efficiency (FE) in pigs. If this is the case, there is huge potential to manipulate the gut microbiome to improve pig growth and FE, which would have considerable economic and environmental benefits. This approach is also particularly timely, given current/impending restrictions on the use of in-feed antibiotics and therapeutic levels of zinc oxide, especially in Europe. Due to these economic, environmental and regulatory drivers, and also due to advances in DNA sequencing technologies, a number of studies have been published recently which investigate the link between the intestinal microbiota and growth, body composition and FE in pigs. Here, we will summarise only data from next-generation sequencing studies. However, even within these studies, it should be borne in mind that diverse methods can be employed for structural and/or functional analysis of the pig gut microbiota [2]. This review will: (1) Summarise the evidence for a link between the gut microbiome and production traits, and attempt to identify common growth- and FE-associated bacterial taxa and functional pathways; (2) outline possible mechanisms by which the gut microbiome might impact growth and FE; (3) suggest ways in which the knowledge accumulated to date can be used to inform strategies to manipulate the gut microbiome to improve growth and FE for commercial pig producers; and (4) outline challenges associated with these strategies. In order to gain an insight into the microbiota composition along the diverse sections of the porcine gastrointestinal tract (GIT), readers are referred to recent reviews/studies in this area [1,2,3,4]. 

## 2. Evidence for a Link between the Pig Intestinal Microbiota and Pig Growth, Body Weight and Body Composition 

**Figure 1:**
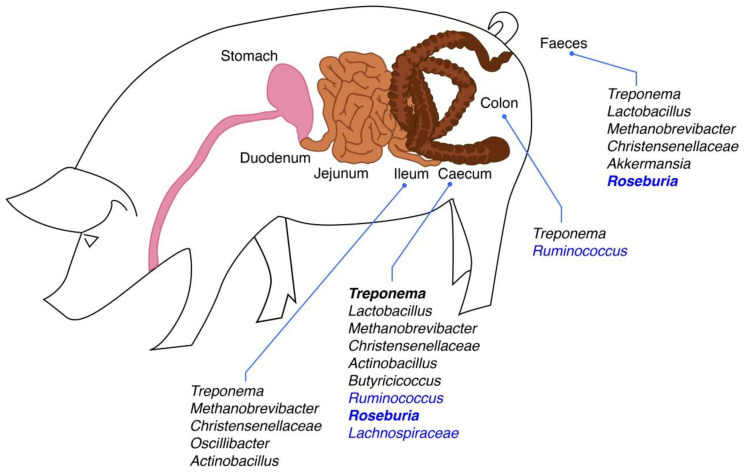
Summary of feed efficiency-associated bacterial taxa in pigs. There are some conflicting data for the taxa in blue, and the taxa in bold are also associated with increased body weight and/or leanness.

Evidence of the link between the intestinal microbiota and pig growth and body composition traits is mainly derived from studies comparing microbiota diversity and composition in pigs ranked as extreme based on these production parameters. We have summarised findings for bacterial diversity and differentially abundant taxa from two studies in which these data were available (Table 1). Data on correlations of taxa with growth and body composition traits from other studies are summarised in the text. Figure 1 also summarises some common findings. Although all are sequence-based, readers should be mindful that the studies summarised here have used a range of methods for structural and/or functional analysis of the microbiota, and these methodologies have been detailed in Table 1 and/or in the text. Due to the enormity of the available data, the focus in this review will mainly be on common findings across studies, as it is beyond the scope of a single review paper to discuss all pig growth-associated taxa. 

Higher bacterial diversity in the GIT, including that of pigs, is generally seen as favourable. Han et al. [5] found higher diversity within the faeces of heavier compared to lighter pigs at 9 weeks of age (the one time point evaluated) and samples clustered according to body weight (BW) on principal component analysis (PCA) plots. Although α-diversity was not measured in pigs ranked on back fat depth in the study conducted by Yang et al. [6], samples also clustered separately on PCA plots, albeit only caecal samples taken at day 300 and not ileal or jejunal samples from the same time point. However, another study found that α-diversity was negatively correlated with both body fat and average daily gain (ADG) [7].

Not surprisingly, in both of the studies represented in Table 1, the bacterial profile differed according to phenotype. Han et al. [5] observed a lower faecal relative abundance of *Bacteroides* and *Anaerotruncus*, two genera that contain pathogenic species [8,9] but that were, however, associated with obesity in humans [10,11], in the heavier pigs. Functional predictions with PICRUSt showed a concomitant reduction in genes related to the nucleotide-binding oligomerisation domain-like receptor (NLR) signalling pathway, which is associated with induction of a host immune response, which is energy-demanding. Hence, reduced activation of this bacterial pathway, possibly due to lower pathogen levels, could explain the improved growth in these animals. Yang et al. [6] also found potential pathogens (*Escherichia coli* and pathogenic species of *Clostridium*) to be less abundant in the ileum and caecum of leaner pigs, a phenotype which is more desirable. Furthermore, *Escherichia/Shigella/Brenneria* had negative correlations with ADG and carcass weight in a 16S rRNA gene sequencing study conducted by Torres-Pitarch et al. [12]. Yang et al. [6] related the lower pathogen abundance to reduced intestinal inflammation, as bacterial inflammation-related pathways determined by metagenomic sequencing (MAPK signalling, endocytosis, antigen processing) were less abundant in the leaner pigs (Table 1).

In agreement with the findings of Han et al. [5], Mach et al. [13] showed via pyrosequencing of the V3–V4 region of the 16S rRNA gene, that faecal abundance of *Bacteroides* was negatively correlated with BW. This could be due to the fact that this genus contains some species that are opportunistic pathogens, as outlined above, thereby leading to diversion of energy and nutrients towards an immune response and away from growth. However, Yang et al. [6] found a number of *Bacteroides* species to be enriched in the ileum and caecum of leaner pigs (Table 1). The latter suggests a beneficial role for members of this genus within both the small and large intestine. This could be due to their ability to degrade complex dietary carbohydrates but also host N-glycans found in mucus at the gut mucosa [14], which may provide them with a growth advantage with lower feed intake of the host. *Lactococcus* was also less abundant in the faeces of heavier pigs in the Han et al. study [5], which is in agreement with the findings of Torres-Pitarch et al. [12], who showed that *Lactococcus* (albeit in the ileum) was negatively correlated with ADG and carcass weight. The reason for this negative association is not known, as other lactic acid bacteria, most notably *Lactobacillus*, are associated with beneficial effects in the gut and better FE, as outlined in the next section. 

Conflicting data have also been obtained for *Prevotella*, a genus considered a core member of the gut microbiome of pigs [15,16]; on the one hand, it was less abundant in the faeces of heavier pigs [5] but Mach et al. [13] found it to be positively correlated with BW. They, along with others [17], also showed that animals belonging to a *Prevotella*-dominated cluster had higher post-weaning BW and ADG. This was explained by the fact that *Prevotella* plays an important role in the breakdown of complex dietary plant-derived polysaccharides, thereby making otherwise indigestible substrates available to the host, with the resultant production of short-chain fatty acids (SCFA) which are absorbed as an energy source. In support of the beneficial role of this genus in the pig gut, Yang et al. [6] found a number of *Prevotella* species to be enriched in the ileum and caecum of lean pigs versus those with high levels of back fat, with the concomitant enrichment of glycoside hydrolase and glycan (polysaccharide) degradation pathways, as determined by metagenomic sequencing. However, the opposite was found in a 16S rRNA gene (V4) sequencing study conducted by Lu et al. [7], in which *Prevotella*-enriched enterotypes, albeit in the faeces, were associated with increased back fat. Interestingly, data on the role of *Prevotella* as a determinant of FE in pigs are also conflicting (see next section). *Treponema* is another genus that was higher in abundance in the caecum of leaner pigs [6], and a 16S rRNA gene (V4) sequencing study by Bergamaschi et al. [18] also found it to be associated with growth and fatness parameters.

The differential abundance of metabolic pathways apart from those outlined above in pigs ranked according to production traits could further justify differences in the host phenotype. For example, genes related to the degradation of xenobiotics (toxic substances, i.e., dioxin, xylene and benzoate) were predicted by PICRUSt to be more abundant within the faecal microbiota of heavier pigs [5]. However, conversely, metagenomic sequencing found that benzoate degradation pathway-related genes were less abundant within the caecal microbiota of leaner pigs [6]. The lean phenotype of these animals may, however, be explained by the lower abundance of pathways related to bacterial metabolism (pyruvate, propanoate, cysteine and methionine metabolism) and nutrient sensing (flagellar assembly, chemotaxis, two-component system and transporters) and the higher abundance of protein digestion and absorption-linked genes. Many of the taxa associated with high levels of intramuscular fat, a desirable meat quality trait, have a role to play in the metabolism of diet-derived nondigestible polysaccharides and amino acids [19]. Predicted functional analysis using PICRUSt agreed with this, as it demonstrated that bacterial pathways related to carbohydrate metabolism, energy and amino acids, cell motility, and membrane transport were associated with intramuscular fat content [19].

Looking at other 16S rRNA gene sequencing studies not represented in Table 1, other bacterial taxa commonly associated with increased BW and leanness include *Roseburia* (another core member of the pig gut microbiome [16]) in the caecum [12,15] and faeces [20], probably due to its role in carbohydrate degradation and as a butyrate producer [21]. However, conversely, it was less abundant in leaner pigs in the Yang et al. study [6]. *Mitsuokella* in the faeces has also been associated with increased growth [13,17], although caecal abundance of this genus was negatively correlated with ADG and carcass weight in the Torres-Pitarch et al. study [15].

Some of the studies referred to here suffer from low numbers of pigs, as phenotypic extremes are selected. However, Bergamaschi et al. [18] used bacterial composition data generated by Lu et al. [7] (via 16S rRNA gene sequencing) from rectal swabs taken at three time points from 1028 pigs. Due to the massive amount of data obtained, it is not possible to summarise it in Table 1. They found 334 operational taxonomic units (OTUs) that were associated, both positively and negatively, with growth and fatness across time points. The higher abundance genera within these ADG- and backfat-associated OTUs were identified as *Clostridium*, *Prevotella*, *Lactobacillus*, *Eubacterium*, *Ruminococcus*, *Streptococcus*, *Bacteroides*, *Treponema*, *Coprococcus* and *Faecalibacterium*. Although the authors do not state if the associations were positive or negative, looking at the supplementary material, it varied within each of these genera, depending on the OTU and the time point. Many of these genera have already been discussed in this section as having an association with pig growth and/or body composition, and most have a role in polysaccharide degradation and/or amino acid metabolism. Most of the relationships occurred during the finisher period. This is in agreement with the findings of Lu et al. [7] who found that enterotypes identified earlier in life (at weaning) did not impact either body fat or ADG. Moreover, Maltecca et al. [22] found, using 16S rRNA (V4) gene sequencing, that the gut microbiome of pigs could be used to predict growth and body composition parameters, particularly fatness traits, but mainly when sampled later in life.

Overall, the studies reviewed in this section serve to highlight the challenges associated with identifying bacterial taxa associated with pig production traits. One of these is the within-study variation in data from different gut locations and pigs of different ages. Variations are also evident across studies, most likely due to differences in breeds, environmental conditions and diet. In addition, even within a study, some taxa are both negatively and positively correlated with the same trait, for example, intramuscular fat in the 16S rRNA gene sequencing study by Fang et al. [19]. This is most likely due to the study’s lack of identification of taxa to the species level, an issue commonly encountered across the studies reported here due to the lack of use of shotgun metagenomics.

## 3. Evidence for a Link between the Pig Intestinal Microbiota and Feed Efficiency

As with pig growth and composition traits, evidence of a link between the pig gut microbiota and FE comes from association studies in pigs ranked on measures of FE: Either feed conversion ratio (FCR) or residual feed intake (RFI). However, more studies have been conducted on FE than growth/body composition, making it difficult to summarise all available data. Nonetheless, we have attempted to do so for findings on bacterial diversity and differentially abundant taxa (Table 2; Figure 1), with additional data from other studies summarised in the text. As outlined in the section above, these types of studies generate huge amounts of data; in some cases, the data were given in the paper; in other cases, we have had to summarise raw data available in supplementary files; and for other studies, we have used the authors’ summary. As with the previous section, the focus will mainly be on common FE-associated taxa identified across studies, and the different methods used for structural and/or functional microbiome analysis are detailed in Table 2 and/or in the text. It should be noted that most studies sampled the large intestine only, with a few exceptions [23,24,25], in which small intestinal samples were also included.

First, a number of studies demonstrated greater bacterial diversity in the ileum [24], caecum and colon [25] of more feed-efficient pigs at the finisher stage. However, others have seen no effect of FE rank on α-diversity indices [23,26,27]. Some studies have, however, observed clustering of caecal samples on the basis of FE [27,28]. This is substantiated by differences in taxonomic composition here, as well as in other parts of the GIT. 

In general, across studies, the majority of FE-associated taxa are more abundant in more feed-efficient pigs (Table 1). These include bacteria that play a role in nutrient processing and energy harvest for the host. Many are involved in carbohydrate degradation, particularly the breakdown of plant-derived polysaccharides, with the resultant SCFA used as an energy source for the pig and some capable of exerting anti-inflammatory effects. These polysaccharide degraders include *Christensenellaceae* which was enriched in the faeces [23], ileum [25] and caecum [28] of more feed-efficient pigs and also found in higher abundance in leaner humans [29]. *Treponema* can also be included here as it is correlated with crude fibre digestibility in pigs [30]. Although members of the genus can be pathogenic, as well as commensal [31], it was more abundant in the ileum [23], faeces [24,32], caecum [25,27] and colon [25] of more feed-efficient pigs. This is also backed up by the fact that pigs clustered into a *Treponema*-dominated enterotype tended to have better FE than those clustered into one dominated by *Prevotella* [32]. *Methanobrevibacter* is a methanogen that is positively correlated with fibre digestibility in pigs [30] and is associated with leaner phenotypes in humans [33]. It was found in higher abundance in the ileum [23], faeces [24] and caecum [27] of more feed-efficient pigs. It was one of only seven genera found to be FE-associated across two geographic locations and/or across two batches of pigs within one location [24]. *Actinobacillus* can also be considered in this category of carbohydrate degraders; it was increased in the caecum and ileum of more feed-efficient pigs [23,24], and high levels in the caecum have been associated with polysaccharide fermentation [34].

Some of the bacteria associated with better FE across studies ferment a range of substrates and are particularly associated with butyrate production. These include *Ruminococcus* (a core member of the pig gut microbiome [16]), enriched in the caecum [25,27,28] and colon [25,26]; *Butyricicoccus* in the caecum [27,28]; *Roseburia* (also a core member) in the faeces [24] and caecum [27,28]; and *Lachnospiraceae* in the caecum [25,28]. Other taxa found to be FE-associated are linked with better gut health and disease prevention, consistent with better overall health in more feed-efficient pigs. For example, *Oscillibacter*, which produces anti-inflammatory metabolites and has been used as a probiotic [35], has been found to be more abundant in the ileum [23] and faeces [32] of pigs ranked as more feed-efficient. This is in agreement with the findings of Tan et al. [28] who showed that *Oscillibacter* was strongly associated with high FE in pigs. *Akkermansia* was also enriched in more feed-efficient pigs, albeit only in the faeces [24,32], and was exclusively found in low-RFI (more feed-efficient) pigs in the faeces at weaning and in the ileum in one study [24]. This genus is considered a ‘next-generation beneficial microbe’, at least for humans, with several studies reporting its ability to impact glucose and lipid metabolism and to reduce intestinal inflammation, mainly due to its ability to maintain/restore gut barrier function [36]. *Lactobacillus* spp., considered one of the core genera of healthy pigs and commonly used as probiotics [37], are also consistently enriched in the caecum [26,27,38] and faeces [32] of more feed-efficient pigs across studies. *Lactobacillus* in the caecal digesta was also correlated with low RFI (better FE) in another study [23]. They were also among one of only four faecal-derived genera found to be positively correlated with improved FE in a 16S rRNA gene sequencing study conducted by Bergamaschi et al. [20]. 

The role of some other genera enriched in more feed-efficient pigs across studies, however, is unclear, for example: *Paludibacter* in the caecum [24,27]; *Alistipes* in the faeces [24] and caecum [27]; and *Blautia* in the ileum [24] and caecum [27]. In addition, *Eubacterium* which is generally FE-associated, i.e., enriched in the caecum [25,28] and colon [25] of pigs, ranked as feed-efficient, although the opposite was true in one study with *Eubacterium coprostanoligenes* less abundant in the caecum of more efficient pigs [25]. Interestingly, *Blautia* and *Eubacterium* were amongst only four genera in the faeces found by Bergamaschi et al. [20] to be positively correlated with FE, and the former is another of the genera considered as core within the pig gut microbiota [16].

Other genera known to be polysaccharide degraders are also most likely associated with improved FE, but conflicting data have been obtained for them. For example, *Bacteroides*, which is associated with host N-glycan breakdown [14], was enriched in the faeces [23] and caecum [25] of more feed-efficient pigs but was less abundant in the caecum [25,27] and colon [25] of feed-efficient pigs in other studies. This could be due to the fact that *Bacteroides* are capable of utilising host-derived substrates such as mucins, as alluded to above, and therefore have a growth advantage in feed-efficient animals that typically have lower feed intake than their less efficient counterparts. *Clostridium* sensu stricto was also more abundant in the faeces [23] and caecum [25] of more feed-efficient pigs, as were various species of *Clostridium* (*boltea*, *clostridioforme*, *saccharolyticum*, *cellulosi*, *clariflavum*) in either the ileum [24], caecum [25,28] or faeces [32]. This is most likely due to the polysaccharide-degrading ability of this core genus [30]. However, reports of lower abundance in the faeces [23] and caecum [27] of more efficient animals could be related to the fact that some species are enteric pathogens [39], thereby causing a more energy-demanding immune response, as described in more detail below. 

Conflicting data were also observed for some of the polysaccharide-degrading butyrate producers referred to above. While they are undoubtedly linked with better FE, it should be noted that *Lachnospiraceae* and *Roseburia* were less abundant in the colon and ileum of less efficient pigs, respectively [25], and *Ruminococcus* was associated with poorer FE, but only in one cohort in the study conducted by McCormack et al. [24]. This could be due to the fact that these genera are competing with the host for nutrients (see section on microbial mechanisms below). Another taxon for which conflicting data were obtained was *Erysipelotrichaceae*, a bacterial family associated with host lipid metabolism and linked to inflammation [40]. On the one hand, it was enriched in the caecum of more efficient pigs [24,28], and on the other, faecal abundance was associated with poorer FE [24]. Lastly, from 16S rRNA gene sequence data, Weishaar et al. [41] identified OTUs of *Oscillibacter*, *Prevotella*, *Corynebacterium*, *Lachnospiraceae*, *Anaerovibrio* and *Clostridia* as having ‘strong effects’ on feed efficiency traits, many of which have been discussed here for their potential to impact FE. However, as is the case in some other studies, they do not specify if these are positive or negative effects.

Other bacterial taxa were consistently found to be less abundant within the gut microbiota of pigs ranked as feed-efficient across studies, although there were much fewer of these and conflicting data were obtained for all. Some of these taxa associated with poorer FE included polysaccharide degraders. For example, *Prevotella* was generally less abundant in more feed-efficient pigs, i.e., in the caecum [27,28] and faeces [32], and only one study found higher abundance in more efficient pigs, i.e., in the colon [25]. It is possible that this genus could be competing with the host for nutrients. The findings outlined above are corroborated by those of Yang et al. [30] who found that pigs clustered into a *Prevotella*-dominated enterotype tended to have poorer FE. Furthermore, a comparable analysis of species within the caecum of low- versus high-FE pigs showed that *Prevotella* could be considered a potential biomarker for the low-FE group [28]. It could be that more substrate was available in the caecum of poorly feed-efficient pigs, due to their inability to efficiently digest ingested feed. With more fermentable substrate entering the caecum, this may have given *Prevotella* a growth advantage. Hence, *Prevotella* abundance could be an effect of poorer FE and not the cause. *Faecalibacterium* was also lower in abundance in the faeces of more feed-efficient pigs [24,30] but was FE-linked in the caecum in the Quan et al. study [27]. Linking this genus with poor FE, however, is at odds with the fact that it is a butyrate producer considered to have anti-inflammatory activity and an involvement in energy harvest, and that its only known species, *Faecalibacterium prausnitzii*, is a ‘next generation’ probiotic [42]. It was, however, found by Bergamaschi et al. [20] to be negatively correlated with FE. As a bacterium that is mostly cross-fed by lactate producers, the question arises whether the abundance difference in *Faecalibacterium* was an effect of differences in abundances of the primary metabolite producers and merely indicated changes in intestinal substrate availabilities. *Streptococcus* was generally less abundant in more feed-efficient pigs, i.e., in the faeces [23], caecum [24] and colon [25]. This could be related to the fact that some species are pathogenic [40], potentially increasing low-grade inflammation of the gut mucosa. However, the fact that *Streptococcus* was also found to be enriched in the ileum [25] and faeces [32] of more feed-efficient pigs is most likely related to their purported beneficial role in the gut, which appears to be mainly due to their ability to produce lactic acid and antimicrobials and less to their potential as pathogens [43,44,45].

Apart from the bacterial taxa discussed above, some pig gut microbiota analyses identified differentially represented taxa, for which conflicting data were obtained across studies, whose role in the pig intestine is not fully established. For example, *Mitsuokella* in the ileum was FE-associated, but in the faeces, it has been associated with poorer FE [24]. Another taxon, vadin BB60, was enriched in the faeces [23,24], ileum and caecum [24] of more efficient pigs and was correlated with low RFI (better FE) in the faeces [23], but was conversely less abundant in a different cohort of feed-efficient pigs in the McCormack et al. study [24]. 

Overall, it appears that bacterial taxa involved in nutrient processing and energy harvest, as well as those associated with anti-inflammatory effects and improved gut health, are enriched in more feed-efficient pigs, while potential pathogens are less abundant. These findings are backed up, at least to some extent, by functional analyses/functional predictions of the gut microbiome of poorly versus highly feed-efficient pigs. The only exception is the Vigors et al. study [38], which found no FE-associated differences in PICRUSt-predicted bacterial pathways in the colon. While it is difficult to summarise all of the findings presented in Table 2, some general observations can be made. For example, predicted/actual bacterial pathways involved in carbohydrate and lipid metabolism are generally enriched in more feed-efficient pigs. Interestingly, while the study by Quan et al. [25] is in agreement with this, they found that PICRUSt-predicted pathways related to protein metabolism were enriched in the colon of less feed-efficient pigs. In a later shotgun metagenomic study by the same authors, functional annotation of the microbiome revealed that pathways related to the metabolism of proteins, nucleotides, cofactors and vitamins, to monosaccharide or energy transportation, and to some antibiotic resistance genes (ARG) were increased in abundance in more feed-efficient animals, while other ARG were reduced [27]. They hypothesised that certain ARG could play an important role in selecting for a more efficient energy-harvesting bacterial community. Likewise, using shotgun metagenomics, Tan et al. [28] found that certain ARG were differentially abundant in poorly versus highly feed-efficient animals. This suggests a role for ARG in shaping gut bacterial communities to influence FE. 

Tan et al. [28] also proposed that microbiota impact host FE mainly via the transport pathways of a range of substrates, including lysine, glycan, and ornithine, which are used for protein synthesis. Similarly, using functional metagenomics, Yang et al. [32] also found that pathways related to nitrogen and amino acid metabolism and transport systems were linked with FE. Using PICRUSt, McCormack et al. [23] also found an increased abundance of pathways predicted to be involved in the biosynthesis of amino acids (phenylalanine, tyrosine, tryptophan, valine, leucine, isoleucine) and metabolism of C5-branched dibasic acid, terpenoids and polyketides in more feed-efficient pigs, suggesting improved metabolic capabilities in these animals. However, they found that predicted genes encoding the phosphotransferase system (PTS), a bacterial sugar transport system, were increased in the ileum of less efficient pigs and suggested that this could be due to higher bacterial energy uptake, leaving less sugar available for animal growth. Interestingly, Yang et al. [32] found the same in the faeces of less feed-efficient pigs. However, as these were functional predictions for the large intestine, it may also be feasible that due to lower digestive capacity or increased substrate availability caused by higher feed intake, the digestive capacity in the small intestine of less feed-efficient animals was surpassed. This may have resulted in more substrate (i.e., protein and carbohydrates) entering the large intestine, leading to a greater degree of fermentation and respective functional bacterial gene abundances in these intestinal segments. Pathways related to bacterial chemotaxis and flagellar assembly were also increased in the more feed-efficient group in the Tan et al. study [28], and these are involved in nutrient sensing, as outlined in the previous section. In the McCormack et al. study [24], although none of the differentially represented PICRUSt-predicted microbial pathways were common across all geographic locations, most of the predicted pathways enriched in more feed-efficient pigs were related to core metabolism, including carbohydrate, energy, and nucleotide metabolism, in agreement with the findings of the other studies discussed here. On another note, the finding by Quan et al. [25] of decreased abundance of PICRUSt-predicted bacterial pathways relating to infectious diseases in more efficient pigs could be indicative of a lower pathogen load in these animals, potentially explaining the better FE. 

Overall, some bacterial taxa are consistently identified across studies as being FE-associated, as summarised in Figure 1, and these could serve as potential biomarkers for FE in pigs. However, as for growth and body composition traits, data are conflicting in some cases. As outlined above, this is most likely due to differences in the animals used and conditions encountered across studies. However, even when genetic, nutritional and management effects were minimised in a study by McCormack et al. [24], none of the 188 RFI-associated taxonomic differences found were common to all geographic locations/batches within a location. Hence, the rearing environment, which includes factors such as maternal influence and herd health status, appears to impact intestinal microbiota more than FE. Similarly, other studies investigating the link between the gut microbiome and FE have found environmental factors to have a huge influence on gut microbiota composition [26,32]. The other issue with the McCormack et al. study [24] and many others which have attempted to identify microbial biomarkers of FE is that a multitude of FE-associated taxa are often identified, some of which are only minor representatives of the pig gut microbiota and some for which a clear role has yet to be elucidated. 

## 4. Potential Mechanisms by Which the Gut Microbiome Impacts Growth and FE in Pigs

It has been estimated that ~10% of the host’s transcriptome, including immunity, cell proliferation and metabolic pathways, is microbially regulated [46]. Therefore, any variation in abundance, composition and metabolic activity of the gut microbial community, as elaborated above, causes changes in the crosstalk with the host animal. As already alluded to in the two previous sections, microbial signalling is not restricted to the gut but impacts systemic nutrient metabolism and brain functioning. Experimentally, it is often difficult to distinguish the microbial cause-and-effect relationships in relation to pig growth and FE, especially when feed-efficient and less feed-efficient animals were identified within a population at the end of the experiment. Organ samples (e.g., tissue from the gut, liver, brain, etc.) can only be collected once during the life of a pig, whereas faeces represent the ‘end product’ of digestion and fermentation processes in the gut. Using pigs from breeding lines with diverging growth and FE may provide a deeper insight into the mode of actions, assuming that pigs show similar FE and growth behaviour throughout their life. There is a growing body of evidence that differences in host metabolism and metabolic rate are strong contributors to the variation in FE in pigs, e.g., [47,48]. Feed-efficient pigs were repeatedly shown to have improved anti-oxidant, metabolic and cellular repair capacities in the mitochondria of various organs including muscle, liver and gut [47,48,49,50]. Variation in digestive efficiency (e.g., activity of digestive enzymes) [38,48,50,51] affects the quantity of nutrients that the host animal can derive from the diet. Simultaneously, host digestion and secretions modulate the gut microbiota composition and fermentative activity by altering substrate availability in the various gut regions, which is an important factor for FE- and growth-related taxa abundance in the large intestine. Despite advances in analytical techniques, especially in –omics technologies, the link between diet, microbiome composition and metabolite production and their reciprocal interaction with the host is still difficult to predict due to the many potential influencing factors and methodological limitations [52]. Despite these limitations and inconsistencies between studies, as mentioned above, there is a consensus that the gut microbiota composition and functional activity may actively contribute to FE- and growth-associated differences in host physiology and functioning [48,50]. 

The gut microbiota interacts with the host via the production of primary and secondary fermentation metabolites (e.g., SCFA, medium- and long-chain fatty acids, biogenic amines, vitamins and antimicrobials), degradation of nutrients which are otherwise indigestible for the host (such as dietary fibre), competition for nutrients (especially in the upper digestive tract), bile acid metabolism, and neurotransmitter production [53]. The host gut, in turn, recognises microbial activity via different routes, such as microbial metabolite sensing via G-proteins (GPR) [54] or receptor recognition of microbiota-associated molecular patterns (MAMP) on the microbial cell surface [55]. While SCFA contribute to the pig’s energy supply when absorbed and exert anti-inflammatory properties, the activation of pathogen-recognition receptors (PRR) expressed on the host mucosa and immune cells by microbial surface antigens can trigger a costly upregulation of the gut mucosal immune response, thereby diverting energy and nutrients from growth [53]. Sequential differences in inter- and intra- gut region conditions largely shape the establishment of the specific gut microbiota, both taxonomically and functionally. These differences include variations in available substrates for growth, pH, redox potential, digesta transit time and antimicrobial secretions, which eventually have consequences for gut microbiota–host interactions [53]. However, only a few of these mechanistic pathways have been investigated in detail in relation to growth and FE in pigs to date. Results on FE- and growth-related changes in the gut microbiota are often separately presented in the literature, without integrating the associated microbial communities with host data, except for feed intake and growth. Another drawback when trying to associate FE- and growth-related gut microbiota–host interactions is that the gut microbiota and host physiology are often investigated in different gut segments. It is obviously unachievable to investigate all inter- (i.e., gut segment) and intra-region (i.e., lumen versus mucosa) differences in the microbiota composition along the GIT and relate them to the host mucosal and systemic response. However, although faeces allow multiple samplings from the same pig and the microbiota composition is relatively similar to the distal regions of the large intestine [56], it hardly represents microbial action and interaction in the stomach, small intestine or proximal large intestine. 

### 4.1. Fermentation of Dietary Substrates

The host diet is the primary substrate source for the digesta-associated microbiota; therefore, nutrient flows are important to consider when interpreting FE- and growth-associated variation within the gut microbiome. Today’s diets for fattening pigs are energy- and nutrient-dense with a low fibre content in order to achieve maximum BW gain. Due to this, the majority of the diet is digested and nutrients are absorbed in the small intestine, whereas a smaller amount of dietary residuals pass into the large intestine. As the entire porcine GIT is colonised by a complex and diverse microbiota, microbes compete with the host animal for nutrients as proximal as the stomach, expressing a plethora of exogenous and endogenous enzymes and transport systems for the degradation and uptake of major and minor dietary nutrient fractions. The extent to which the gut microbiota contributes to the apparent ileal and total tract digestibility coefficients can hardly be estimated from the standard approaches, for which inconsistent results for FE-related variation have been reported. Accordingly, the digestive efficiency did not differ between pigs divergently selected for RFI in some studies [57,58,59], whereas others reported improved apparent total tract digestibility of dry matter with low RFI [38,60]. Especially, the contribution of the gut microbiota to the degradation of easily digestible fractions, such as starch, sugar and protein, cannot be separated using digestibility marker- or balance-based approaches. Digestibility coefficients for dietary fibre fractions, such as neutral detergent fibre (NDF), are better indicators for FE-associated differences in microbial activity; however, they only cover hemicellulolytic and cellulolytic activity and largely miss FE- and growth-associated differences in the metabolic activity of saccharolytic and proteolytic species. In this regard, pigs from a low-RFI line showed a higher apparent ileal NDF digestibility when fed a high-fibre diet compared to the respective high-RFI line, indicating improved fibre utilisation and enhanced fermentative activity in the upper digestive tract [59]. This was accompanied by lower concentrations of primary fermentation metabolites, i.e., total SCFA, acetate and butyrate, in caecal digesta of pigs from the low-RFI line compared to pigs of the high-RFI line, as well as shifts in the molar proportions of acetate to propionate towards more propionate in the caecal and colonic contents of the low-RFI line [59]. Unfortunately, changes in the digestive capacity and luminal SCFA were not linked to specific taxonomic abundances in this study. 

Correspondingly, Reyer et al. [50] reported an increased abundance of dietary fibre-fermenting taxa, such as *Rothia*, *Subdoligranulu*, *Leeia*, *Cellulosilyticum*, and the unidentified rumen bacterium RFN43, in low-RFI pigs at slaughter weight, allowing a more efficient utilisation of dietary fibre in ileal and caecal segments. These observations were supported by findings for colonic SCFA concentrations in fattening pigs selected for RFI after 115 days on trial [38]. While having a similar apparent ileal digestibility of dry matter, low-RFI pigs were further characterised by a greater apparent total tract digestibility of dry matter, indicating higher fermentative action in the large intestine compared to their high-RFI counterparts [38]. Although the authors investigated the expression of a plethora of genes related to host digestion and glucose and amino acid uptake in the jejunum of these pigs, the expression of SCFA transport proteins was not measured, to link microbial metabolic activity and effects on the host. In contrast to Vigors et al. [61] who found lower SCFA concentrations in the caecal content of high-RFI pigs, Metzler-Zebeli et al. [62] reported equal caecal SCFA concentrations in low- and high-RFI pigs. However, for the daily production of SCFA, feed intake needs to be considered. Therefore, a higher daily SCFA production in the caecum, as also reported by Montagne et al. [59], for high-RFI pigs may be assumed, which may be supported by the trend for increased expression of monocarboxylate transporter 1 and signalling genes (glucagon-like peptide-1 and peptide YY) in high- compared to low-RFI pigs [62]. After gut mucosal uptake, the absorbed SCFA contribute to energy homeostasis (e.g., propionate) and de novo lipogenesis (e.g., acetate) in the liver. However, due to the different metabolic fates of the individual SCFA, this may have consequences for body composition, leading to the FE-related variation in physiological traits and potentially FE of the host [63]. Therefore, it can be speculated whether the smaller acetate:propionate ratio found in the large intestinal content of low-RFI pigs [59] may have contributed to a leaner carcass because less acetate would mean less precursors for hepatic lipogenesis. Likewise, Vigors et al. [38] reported less acetate in the colonic content of low-RFI compared to high-RFI pigs. Propionate is converted to glucose in hepatocytes, and may be used as an immediately available energy source or stored as glycogen in liver and muscles. Following this reasoning, pigs selected for a low RFI exhibited improved carcass merit in having less back fat and belly fat and more lean mass than pigs selected for a high RFI [64]. Moreover, propionate signals with greater strength via the G-protein receptor (GPR)-41 than acetate not only at the gut mucosa but also on immune cells and adipocytes [54]. In mice, this receptor was required for the SCFA-mediated release of leptin from adipocytes [65], potentially supporting a role of this receptor in satiety [54]. In this context, Reyer et al. [50] reported higher serum leptin levels in more feed-efficient pigs, which may be contradictory to the lower carcass fat observed by others for pigs of low RFI [64], but may have been mediated via GPR-signalling due to altered intestinal SCFA generation. Integration of SCFA data with those of the mucosal gene expression in the caecum of low- and high-RFI pigs indicated a certain signalling by the luminal SCFA [62], indicating a potential signalling via activation of GPRs [54]. As a result of the GPR signalling, SCFA can enhance host insulin sensitivity by promoting glucagon-like peptide-1 secretion [54] and satiety by stimulation of peptide YY secretion [66], thereby potentially reducing feed intake in low-RFI pigs. Contrary to this reasoning, gene expression results at the caecal mucosa of low- and high-RFI pigs showed the opposite effect, with a lower expression of glucagon-like peptide-1 and peptide YY in low- versus high-RFI pigs [62]. Likewise, Reyer et al. [48] did not find FE-associated differences in glucagon-like-peptide-1 and peptide YY levels in serum but higher leptin, insulin and gastric inhibitory polypeptide in low-RFI pigs, potentially indicating improved utilisation of nutrients with better FE. Unfortunately, the contribution of the gut microbiota could not be deduced from the serum data. 

These results emphasise the importance of complex interactions between nervous, endocrine, and immune processes at the gut mucosa and systemic level which need to be considered as a whole [63] and not separately, rendering it difficult to clearly distinguish between microbiome and host effects. Nevertheless, prediction of enzymatic capacities from caecal and faecal metagenome data of pigs selected for RFI would support an improvement in FE by increased energy-provision and promotion of health through SCFA via the fermentation of dietary polysaccharides and improved utilisation of dietary protein [28,32,62]. These data would also fit with the higher abundance of dietary fibre-fermenting taxa in ileal and caecal digesta of low-RFI pigs in the study of Reyer et al. [50], pointing towards a more efficient utilisation of dietary fibre in these segments.

Enterocytes use single SCFA in a preferential order, with butyrate being the most favoured substrate [67]. Besides undergoing beta-oxidation to acetyl-CoA, butyrate modulates gene expression in the enterocytes with effects on cell proliferation [54,68]. Other SCFA, such as valerate, caproate, propionate and acetate, equally contribute to the ATP generation and influence cell metabolism in enterocytes when butyrate concentrations become low [67,68]. Montagne et al. [59] reported a greater empty colon weight in pigs of a high-RFI line compared to those of a low-RFI line, which may be related to a greater daily production of SCFA available as respiratory fuel for the enterocytes. Accordingly, the higher feed intake in pigs of the high-RFI line may have stimulated SCFA-mediated cell proliferation in the colon, diverting energy and nutrients for growth due to higher basal metabolic demands of the heavier organ. As a gut segment with high fermentative activity, FE-related differences in the daily SCFA production can be expected for the caecum too. However, the empty caecum weight was similar for pigs selected for [51] or from lines of low and high RFI [59]. 

Although information on bacterial long-chain fatty acid production is scarcely available for pigs, it can be assumed that differences in the lipogenetic or lipolytic activity of the gut microbiome may have consequences for carcass composition, i.e., lipid quality [69]. This assumption is supported by the trend of higher abundances of genes related to the biosynthesis of unsaturated fatty acids in low-RFI pigs compared to their high-RFI counterparts [62]. In following this reasoning, the greater percentage of monounsaturated fatty acids in subcutaneous adipose tissue of pigs from a low-RFI line may point in this direction, which was represented by a greater proportion of C18:1 n-9 in the low- compared with the high-RFI line [64]. However, the published data again do not allow for distinction between microbial and host-related lipid metabolism. 

### 4.2. Modulation of Inflammation

Maintaining a competent and alert immune system is metabolically costly to the host, diverting energy and nutrients from other nutrient-demanding processes such as growth, reproduction and thermoregulation [70,71]. Low-grade gut mucosal inflammation caused by bacterial abundance or metabolites may, therefore, be sufficient to redirect energy in low-FE pigs that is otherwise available for growth in high-FE pigs. The host controls the composition of the gut microbiota via its mucosal immune system and diet, whereas simultaneously, the composition of the commensal microbiota actively shapes the host mucosal and systemic immune homeostasis at multidimensional levels [72]. Due to the importance of the gut as the largest immune organ of the body, microbial stimuli for regulating gut mucosal inflammatory responses and the build-up of immune tolerance have been discussed with regards to porcine FE. The innate immune response is considered more metabolically costly than the adaptive immune response [71]. Moreover, the innate immune system develops earlier than the acquired [53] and thus may play a greater role for FE in the young growing pig. Consequently, differences in the activity and responsiveness of the innate immune system to the gut microbiota could have measurable effects on FE. Recent results for the ileal and caecal transcriptome from pigs at slaughter weight also indicated an impact of the acquired immune system that responded more strongly towards the local gut microbiota [50]. Consistent findings in the literature show that, under non-challenge conditions, the impact of the innate immune response for FE variation is small [48,50,51,61,62,73]. However, this may be different under gut immune challenge conditions with bacterial toxins, such as lipopolysaccharide (LPS), in which the gut mucosal immune response seems to be weaker in low-RFI than in high-RFI pigs, e.g., [61]. 

Gut commensals contribute to FE-associated differences in the gut immune response via two components: MAMPs on the bacterial cell surface (acting as a ‘fingerprint’), which trigger direct activation of the PRR; and microbial metabolites, such as SCFA and medium-chain fatty acids, which can attenuate pro-inflammatory responses at the gut mucosa and virulence gene expression in pathogens [53,54]. As a consequence, differences in the presence of commensals result in different activation of PRR at the gut mucosa, whereby the complexity and diversity of the commensal composition seem to be critical for a protective mucosal immunity [72]. The recognition of MAMPs by PRRs (e.g., toll-like receptors (TLR), NLR and C-type lectins) induces intracellular signalling cascades, leading to an inflammatory response, recruitment of phagocytic cells for clearance of pathogens, and mobilisation of professional antigen-presenting cells [53]. These include activation of genes that encode pro-inflammatory cytokines, anti-apoptotic factors, and antimicrobial peptides (for more details, see review of [53]). So far, only a few studies have used transcriptomic approaches to detect differentially expressed immune genes or gene clusters in pigs of diverging FE, e.g., [48,50]. On the other hand, a number of studies have used targeted approaches focusing on the physical and chemical barriers by which the host controls and interacts with the microbiota in the gut lumen, including tight-junction proteins, mucins, as well as cytokine-signalling cascades related to TLR signalling as the best-investigated PRRs [51,61,62,73]. In this context, especially, the LPS-associated activation of TLR4/TLR2 has received special attention due to the role of LPS as a strong immune stimulant [53]. 

Under non-challenge immune conditions, only minimal FE-associated variation in the innate immune response related to TLR and cytokine signalling in jejunal, ileal, caecal and colonic tissues were reported [48,50,51,61,62]. Interestingly, when using a similar experimental design and sampling protocol at two different research (geographic) locations [51], an increased *TLR4* and *TNFA* expression was found for the ileal mucosa of high-RFI pigs, but only in one pig population (Austria) and not in the other (Republic of Ireland). These results pointed towards a different Gram-negative microbial colonisation of the jejunum between the two geographical locations or different responsiveness of the jejunum towards luminal stimuli of low- and high-RFI pigs. The jejunal microbiome composition was not analysed in this study; however, location-specific variation in the ileal, caecal and faecal RFI-associated microbiomes, which were outlined in detail above, may support different microbial colonisation [23]. Hence, RFI-related microbe–host immune responses may depend on the specific pig farm, each creating a unique microbial signature. However, for the understanding of cause-and-effect relationships, these data do not enable us to determine whether differences in microbial colonisation between the low- and high-RFI siblings or host-related differences in hypothalamic feed intake regulation and, thus, digesta flow caused the differences in small intestinal TLR-4 signalling. As the same Austrian pigs showed equal *TLR4* and *TNFA* expression at the caecal mucosa [62], these findings further emphasise the inter-segmental differences in gut-microbiota–host signalling, especially as the caecal microbiota communities in low- and high- RFI pigs largely differed in their abundance of Gram-negative bacteria (e.g., *Campylobacter*), which was assumed to have caused low-grade inflammation via the TLR-4-NF-κB signalling pathway. As the Gram-negative *Helicobacteraceae* and *Campylobacteraceae* were the most abundant families [62], the constant exposure of the gut mucosa to their TLR ligands may have induced a basal state of activation of downstream signalling pathways, tempering potential inflammatory responses [72]. Overall, there was a clearly distinguishable microbiome signature on the caecal innate immune response associated with porcine FE in this study, emphasising that certain mucosal bacteria and microbial metabolites within the caecal content were more associated than others with RFI, mucosal expression of SCFA sensing, and innate immune response genes [62]. For instance, lower mucosal *MUC2* but equal *MUC4* expression in low-RFI pigs compared to high-RFI pigs pointed towards host (FE)-derived differences in mucin synthesis or bacterial degradation associated with the greater *Campylobacter* abundance in low-RFI pigs. *Campylobacter*-modulated host glycan expression following invasion in either low-RFI [74,75] or high-RFI pigs showed a greater and metabolically costly mucosal immune response to the residing caecal microbiota than low-RFI pigs by expressing more *MUC2* in order to counteract mucosal *Campylobacter* invasion [76]. Nevertheless, results did not allow for the determination of whether mucosal bacterial changes were behind the variation in mucosal gene expression or, rather, a consequence of FE-related changes in host physiology or feed intake behaviour [62]. Accordingly, Reyer et al. [48] concluded that many of the differences seen in the transcriptome of small intestinal segments might reflect adaptations of the host to altered gut conditions due to the FE-associated variation in the gut microbiome composition and metabolic activity. 

Although Mani et al. [73] reported similar ileal and colonic barrier function between pigs from low- and high-RFI lines at the gene expression and functional protein level, high-RFI pigs had drastically higher circulating serum LPS than low-RFI pigs, indicating that high-RFI pigs were in an increased inflammatory state. As LPS is a component of the outer cell membrane of all Gram-negative bacteria, the LPS in the serum probably originated from the gut. The low-RFI pigs were characterised by higher activities of alkaline phosphatase in the ileum and liver and lysozyme in the ileum, indicating increased nonspecific LPS-detoxifying components and, hence, an enhanced innate immune response [73]. Although the higher secretion of detoxifying components also diverts energy and nutrients from growth, lower inflammation seemed to be the greater factor for FE in their study. Likewise, Reyer et al. [50] observed FE-associated differences in the ileal immune competence and mucosal microbe-host-crosstalk of non-challenged pigs, including an enrichment of the ‘crosstalk between dendritic cells and natural killer cells’ pathway, and differences in the activity of the complement system, regulation of leukocyte chemotaxis in inflammation, and neuronal nitric oxide synthase signalling, which might alter local blood flow and muscle contraction, in more feed-efficient pigs. Caecal transcriptome data showed a similar mucosal upregulation of humoral and cellular immune responses including the complement system, T-helper cell (Th)1 and Th2 pathways system in low-RFI compared to high-RFI pigs [50]. Under challenge conditions, Vigors et al. [61] demonstrated in an ex vivo challenge model that colonic tissue from low-RFI pigs revealed a lower cytokine expression (including interleukin (IL)-8, IL-1, IL-6, tumour necrosis factor (TNF)-α, interferon(IFN)-γ and suppressor of cytokine signalling (SOCS)-3) than tissue from high-RFI pigs challenged with *E. coli*-derived LPS. These results support the theory that a possible energy saving mechanism exists in the intestinal innate immune response towards immune-stimulating MAMPs in more feed-efficient pigs, leading to a moderation of the immune response towards the local commensal microbiome [61]. 

Lately, the role of SCFA as modulators of the native and adaptive immune system has received more attention [54]. Therefore, differences in the SCFA profile or the total amount of SCFA produced may also contribute to the FE-associated differences in inflammatory responses between low- and high-RFI pigs which appear to be, as with nutritional signalling, mediated via activation of GPRs [54,68]. Due to the higher feed intake, more SCFA are theoretically produced in high-RFI pigs [59]. Consequently, less feed-efficient pigs should benefit most from a potential anti-inflammatory effect of SCFA. The activation of GPRs, such as GPR43, GPR41 and GPR109A, by SCFA triggers mucosal pathways that moderate inflammatory responses and influence epithelial integrity, as well as immunoglobulin A antibody responses and macrophage, regulatory T-cell and dendritic cell activities [54,77]. However, the different binding affinities of the individual SCFAs for the various GPRs need to be taken into account. Especially, butyrate and propionate signal with greater strength via GPR41 and GPR43, respectively, than acetate and exert direct anti-inflammatory and anti-apoptotic effects at the gut mucosa and systemically [54,69]. As a consequence, the smaller acetate:propionate ratio in the digesta of low-RFI pigs [38,59] may have led to a stronger activation of anti-inflammatory pathways than digesta richer in acetate. Moreover, activation of the GPRs is probably self-limiting depending on the receptor density expressed at the gut mucosa, thereby restricting potential benefits of increased luminal SCFA levels. In addition, SCFA, i.e., butyrate, can lower inflammation via inhibition of histone deacetylase expression or function by down-regulating pro-inflammatory mediators, including nitric oxide, IL-6 and IL-12 [78]. These SCFA effects are not limited to the gut lumen, but after absorption, SCFA exert systemic effects through circulating in the plasma, influencing neural activity, the inflammatory response, glucose homeostasis, lipid metabolism, and endocrine hormone and gut hormone secretion, whereby the different GPRs activate different pathways [68]. 

In the gut lumen, SCFA influence the microbiota composition due to their antimicrobial properties, possibly potentiating FE-related differences in the gut microbiome, with consequences for the MAMP-associated mucosal immune activation. In adequate concentrations, SCFA have direct antimicrobial activity against pathogenic bacteria [79,80]. For instance, propionate production from *Bacteroides* mediated resistance to *Salmonella* colonisation [80], which might explain the association of this genus with high FE in some studies. Likewise, the administration of coated butyrate to pigs decreased *Salmonella* shedding and intestinal colonisation [81]. The protective and antimicrobial effects of SCFA are concentration-dependent. If intestinal SCFA levels fall below a certain threshold, the inhibitory effect of SCFA, such as butyrate, on the expression of virulence genes ceases, as shown for enterohemorrhagic *E. coli* and *Campylobacter jejuni* [82]. As this inhibitory action of SCFA has not been sufficiently studied in relation to FE/growth, this may be one mechanism to be explored in more detail in the future in order to determine their contribution to the link between the gut microbiome and host FE and growth. 

## 5. Applying Knowledge on FE- and Growth-Associated Microbiota to Improve Production Traits in Pigs 

One of the goals of research aimed at defining the microbiota differences associated with production traits is to develop strategies to manipulate microbiota composition to promote growth and/or FE. For example, identification of bacterial taxa associated with superior growth and FE in farm animals may offer a direct approach in which these taxa can be used as probiotics in dietary formulations [83]. However, there are considerations that may render the development of suitable FE- and/or growth-enhancing probiotics difficult and, hence, may limit the applicability of a “traditional” probiotic approach. One of these is the inter- and intra-segmental differences in gut microbiota composition [1,2,3,4,16]. As evidenced by the studies reviewed here and as illustrated in Figure 1, most of the knowledge accumulated on FE- and growth-associated bacterial taxa is for the ileum, caecum, segments of the colon and faeces, representing mainly the distal sections of the GIT. In addition, in many cases, the identified bacteria did not belong to the dominating taxa but rather to lower-abundance groups. There is also the lack of consistency across and within studies in terms of identifying taxa that could potentially be used as probiotics. This is illustrated by the research performed by McCormack et al. [23,24] which found that only a few taxa were consistently indicative of low RFI (better feed efficiency) across different geographic locations or replicate batches, as outlined above. These included two uncultured genera, which serves to highlight the fact that the culturability of FE-and growth-associated bacterial taxa is another limiting factor when considering their use as probiotics. This applies not only to those genera that have not been cultured before but also for genera with specific, often unknown, growth requirements. However, recent advances in culturing techniques for members of the gut microbiota may enable their exploitation in the future [84]. 

A bigger challenge perhaps is the fact that many bacteria live in a mutualistic network with other bacteria, being incapable of growing in their absence. A number of intestinal microorganisms, for example, have adapted to utilise the by-products of primary degraders of food sources, in a process known as cross-feeding [85]. Accordingly, species specialising in the breakdown of complex polysaccharides are providers of less complex molecules, which are used by a larger network of bacteria. For instance, growth rates for *Fibrobacter succinogenes* and *Ruminococcus flavefaciens* were enhanced when co-cultured with *Methanobrevibacter* species [86]. Despite this awareness, insufficient knowledge exists on how bacterial populations within the gut communicate, employing small metabolites for signalling with each other and across other communities [85]. 

Therefore, the application of faecal or intestinal transplants may be more promising as the whole microbial community is applied. There is growing evidence for the efficacy of faecal microbiota transplantation (FMT) in treating enteric disease in humans, mainly *Clostridium difficile* infection [87]. Research on FMT as a possible tool to improve health and productivity outcomes in pigs through manipulation of the gastrointestinal microbiome is very recent and, therefore, only limited data are available [88]. However, reprogramming the maternal and/or offspring microbiome to promote FE in pigs by using faecal transplants derived from highly feed-efficient pigs with/without inulin supplementation of offspring did not reproduce the highly efficient phenotype in the offspring [89,90]. On the contrary, the FMT had detrimental effects on lifetime growth. In addition, the gut microbiome composition and predicted functionality were also affected but not as predicted, indicating that FMT, at least the particular regime used, may not be a suitable approach to optimise productivity in pigs. However, if an improvement in growth and/or FE was achieved with FMT, the taxa increased in abundance as a result of the intervention could potentially be exploited as probiotics. For example, five bacterial species were identified as more abundant within the intestinal microbiota of piglets receiving an oral microbiota transfer, which conferred diarrhoea resistance [91]. A subsequent feeding trial showed that pre-weaning oral administration of either the five-strain mix or a *Lactobacillus gasseri* or *Lactobacillus frumenti* strain alone decreased diarrhoea incidence in early-weaned piglets. 

An alternative to the use of probiotics or FMT is to use a more indirect approach whereby specific FE- and/or growth-associated taxa are targeted by dietary means. This could include modification of the composition of the diet and/or supplementation with feed additives such as prebiotics, enzymes, organic acids, etc. While using prebiotics to enhance the growth of beneficial taxa, especially lactic acid-producing bacteria, seems logical to promote gut health, studies are limited as to whether or not prebiotics can specifically promote gut bacteria that are associated with better growth and/or FE. In one study, supplementation of inulin did not affect the growth or FE of pigs but upregulated duodenal genes linked to SCFA absorption [90], being indicative of the fermentation of inulin mostly in the stomach. Due to its high fermentability, only very small amounts, if any, of inulin reach the ileum and large intestine [92] and, therefore, it is not suitable as a means of promoting bacterial taxa associated with better production phenotypes. 

Alternatively, in-feed enzymes could potentially release substrates for specific FE- and growth-associated bacteria within the GIT. For example, the fact that bacterial taxa positively correlated with pig growth (*Lactobacillus kisonensis* and *Roseburia faecis*) were more abundant in pigs fed diets supplemented with a carbohydrase complex could very well have accounted for the improved FE observed in these animals [15]. However, in a sister study, the same enzyme complex promoted the abundance of bacterial taxa that were negatively correlated with pig growth and reduced the abundance of taxa with positive associations with pig growth and caecal butyrate concentration, with a concomitant lack of effects on pig growth and FE [12]. 

Due to the importance of microbial metabolites, e.g., SCFA, in the communication between the gut microbiome and the host, and the link of a smaller acetate:propionate or butyrate ratio in the large intestine with better FE [59], promotion of intestinal propionate and butyrate producers via specific prebiotics (e.g., resistant starch, β-glucan or inulin [21]) or the application of propionate and butyrate or their salts may be feasible dietary strategies to improve porcine FE. The coating of prebiotics or SCFA may be necessary to reach the targeted gut site. Supplementing pig diets with (coated) butyrate has already been used as an efficient strategy to enhance epithelial morphology, homeostasis, and microbiota balance post-weaning [93,94], and has been related to improved growth performance, carcass composition and FE in growing pigs [95]. As pigs of lower FE seem to have a greater response to immune stimuli, increasing the concentrations of anti-inflammatory SCFA (e.g., propionate and butyrate) in the gut may help to moderate an ‘over-shooting’ immune response towards intestinal immune stimuli of the commensal microbiota (e.g., LPS). In this respect, oral administration of propionate has been suggested for the treatment of neurodegenerative disorders in humans, supporting the GPR-mediated anti-inflammatory signalling along the ‘gut–brain axis’ [96].

Another option is to use common FE- and growth-associated bacterial taxa as predictive biomarkers which can be selected for in breeding programmes. Bergamaschi et al. [18] showed that a number of bacterial taxa were moderately heritable, suggesting that pig microbiome composition could be manipulated through selection, in the same way as traits such as fertility and disease resistance. Maltecca et al. [22] concluded that the inclusion of OTU predictors in genomic selection programs had the potential to promote fast growth, while limiting fat deposition. Although another study concluded that it was possible to breed for the microbiota effect on FE, they cautioned that this optimises the microbiota composition for this parameter alone, which may not be optimal for other production traits [41]. This is in agreement with the findings of some of the studies outlined above, whereby some bacterial taxa appear beneficial for growth but not FE and vice-versa. 

With all of the growth- and FE-improving approaches outlined here, the main question is: Which bacterial taxa should be used as probiotics or targeted via dietary supplementation/breeding strategies? However, with different underlying microbiome compositions observed in different investigations and different bacteria related with growth, FE and body composition traits in the various studies outlined above, the selection of microbial targets that can reliably deliver beneficial effects on pig growth and FE from various microbiota is challenging. Nonetheless, from the data outlined earlier, it seems as if certain taxa are emerging as potential targets (Figure 1). These include: (1) *Treponema* in the small and large intestines, which was higher in abundance in leaner pigs and is consistently associated with better FE; (2) methanogens such as *Methanobrevibacter* in the small and large intestines; and (3) *Lactobacillus* in the large intestine, both of which are also commonly associated with better FE. *Treponema* and especially *Lactobacillus* can be considered part of the so-called ‘core microbiota’ of healthy pigs (as reviewed by Valeriano et al. [37]). Other taxa that appear promising as biomarkers for growth and/or FE, such as *Ruminococcus, Roseburia* and *Prevotella*, are also core members of the gut microbiome of commercial pigs [16], but conflicting data have been obtained for them. 

The taxa outlined above could form the basis of a panel of target taxa for manipulation and/or a potential set of microbial biomarkers for FE/growth in pigs. However, the fact that the bacterial taxa potentially driving the differences between good and poor performers were very different across studies presents challenges when aiming to obtain consistent, reliable, microbially driven improvements in FE and growth. It is possible that farm-specific approaches are needed, for example, in a similar way to which personalised medicine is recommended for humans or like farm-specific vaccination against local enterotoxigenic or Shiga-toxin producing *E. coli* strains on farms with a high risk for weaning-associated diarrhoea and oedema disease. This could potentially involve analysing the microbiota of the pigs on a particular farm, with a view to designing appropriate farm-specific targeted strategies. However, the practicality of this approach in commercial pig production settings is questionable. Another issue is that different species within a bacterial genus or even strains within a species can act very differently, so it is likely that we need to look at the OTU level when recommending target taxa, highlighting the importance of shotgun metagenomic studies. Furthermore, when considering dietary manipulation of the gut microbiome, it may take a number of weeks for compositional and/or functional shifts to occur, as previously observed [97]. Moreover, once adapted to the new dietary conditions, this manipulated microbiota composition/function may not persist over time.

Furthermore, the data on the function of individual microbiota members arise from different investigation methods, i.e., sequence-based correlation studies, investigation of whole genome sequence data and culture-based investigations. These diverse investigation methods may very well result in different depictions of the same microbial community. It is also worth mentioning that the function of the microbiota as a whole cannot be considered as the sole sum of the functions of its individual members. Instead, synergistic cooperation between microbial members results in the performance of functions not attributable to a specific member. This had led to the current thinking of the gut microbiome as a functional unit rather than an assemblage of individual microbes. Therefore, an alternative approach would be to focus on core functionality of the microbiome rather than taxonomic nomenclature and to select for production traits based on this. Furthermore, an alternative to the manipulation of microbiota composition would be to modulate overall metagenomic functions of the microbiome (e.g., SCFA production, inflammatory responses) rather than focusing on core taxa per se. Lastly, the studies conducted to date and reviewed here that provide evidence of a link between the pig gut microbiota and production traits are association/correlation studies, and hence, causation cannot be implied. In fact, some specific examples are outlined where increased abundance of certain microbiota members may be a result of improved productivity traits rather than the cause of them. Therefore, the microbial taxa highlighted cannot be interpreted conclusively as determinants for FE/growth in pigs without suitable intervention studies. 

## 6. Conclusions

The potential of the gut microbiota to impact pig growth and FE cannot be ignored. Compositional FE- and growth-associated differences within the intestinal microbiota are commonly found throughout the life of the pig, but mostly at the end of the finishing period. However, these differences can be subtle, occurring for taxa present at low relative abundances and/or whose role within the pig gut microbiome has not yet been fully elucidated. Furthermore, consensus among studies investigating the link between the gut microbiome and growth, body composition and FE in pigs demonstrates the enormous impact of environmental factors on bacterial taxa associated with these production traits. This leads to intra- and inter-study-related differences in the bacterial taxa identified. In addition, cause-and-effect relationships within the gut microbe–host interplay related to pig growth and FE are often not distinguishable. All of these factors present challenges when aiming to identify consistent reliable microbial biomarkers for pig growth and FE.

Nonetheless, as demonstrated by our in-depth review of the literature, certain bacterial taxa have emerged as consistently associated with better production phenotypes, for example, *Treponema* and *Methanobrevibacter* in the small and large intestines, as well as *Lactobacillus* in the large intestine. Others, such as *Bacteroides*, and butyrate producers, such as *Roseburia* and *Ruminococcus*, also appear promising, mainly with regard to their presence in the large intestine and their association with improved FE, although data are conflicting in some cases. In general, the growth- and FE-associated taxa identified here are mostly associated with nutrient processing and energy harvest for the host, but some are related to anti-inflammatory effects and improved gut health. Concomitant enrichment of the bacterial pathways related to carbohydrate and lipid metabolism suggests improved metabolic capabilities in animals with superior growth, body composition and FE traits. Underlying potential microbial signalling routes may involve increased intestinal propionate production and reduced inflammatory response. In conclusion, the growth- and FE-associated bacterial taxa identified in this review could potentially be used as probiotics and/or targeted by dietary or breeding strategies in order to improve FE and/or growth in pigs. However, given the farm-specific differences in growth and FE-associated taxa, it may not be a simple process to select for an ‘ideal’ microbiome profile, and herd-specific strategies may be required. Alternatively, considering that the gut microbiome is a functional unit rather than an assemblage of individual microbes, it may be more appropriate to select for production traits based on core functionality of the microbiome, or the overall functionality of the microbiome could be modulated rather than focusing on core taxa per se. Overall, if successful, the approaches outlined here have the potential to reduce both production costs and the environmental impact of pig production.

## Figures and Tables

**Table 1:** Bacterial diversity within the gut microbiome of pigs ranked on growth/body composition traits and bacterial genera/species and related pathways/genes ^1^ linked with growth and body composition.

Trait (Methodology for Structural and Functional Analysis of Gut Microbiome)	Sample Type (Total Number of Pigs)	Age of Pigs	α- and β-Diversity Effects in Animals with Better Production Traits	Genera/Species More Abundant in Animals with Better Production Traits, i.e., Heavier, Lower Back Fat (Leaner)	Genera/Species Less Abundant in Animals with Better Production Traits, i.e., Heavier, Lower Back Fat (Leaner)	Functional Analysis (↓ = Bacterial Pathways Less Abundant in Animals with Better Production Traits; ↑ = Pathways More Abundant in Animals with Better Production Traits) ^1^	Ref
Body weight (16S rRNA gene sequencing (V4) on Illumina MiSeq + PICRUSt predictions)	Faeces (*n* = 18)	Day 63 of age	Higher α-diversity (ACE, Chao1, and observed OTU indices) for heavier pigs. β-diversity effects at phylum but not genus level	*Anaerococcus Sediminibacterium Butyrivibrio*	*Lactococcus* *Anaerotruncus* ***Eubacterium*** *Bilophila* *Bacteroides* *Prevotella* *Corynebacterium*	↑dioxin, xylene, benzoate degradation ↓Nucleotide-binding oligomerization domain-like receptor (NLR) signalling ↓Alanine, aspartate, glutamate metabolism ↓Novobiocin biosynthesis	[5]
Back fat (16S rRNA gene sequencing (V4) on Illumina MiSeq + metagenomic shotgun sequencing on Illumina HiSeq 2000)	Jejunum (*n* = 8)	~Day 300 of age	α-diversity not measured No β-diversity differences	*Actinobacillus succinogenes* *Cellulosilyticum lentocellum* *Fusobacterium nucleatum* *Haemophilus influenzae* *Mannheimia succiniciproducens*	*Clostridium phytofermentans Lactobacillus johnsonii Escherichia fergusonii*	↑DNA repair and recombination ↑Pyrimidine metabolism ↑Secretion system ↑Lysozyme and chitinase degradation ↑Folate biosynthesis ↑Tropane, piperidine, pyridine alkaloid biosynthesis ↓Nucleotide excision and mismatch repair ↓Pyruvate, propanoate, cysteine, methionine metabolism	[6]
	Ileum (*n* = 8)	~Day 300 of age	α-diversity not measured No β-diversity differences	*Actinobacillus succinogenes* *Cellulosilyticum lentocellum* *Bacteroides dorei* *Bacteroides fragilis* *Bacteroides helcogenes* *Bacteroides salanitronis* *Bacteroides thetaiotaomicron* *Bacteroides vulgatus* *Bacteroides xylanisolvens* *Barnesiella viscericola* *Prevotella dentalis* *Prevotella denticola* *Prevotella ruminicola*	*Chamaesiphon minutus* *Clostridium difficile* *Clostridium sordellii* *Escherichia coli* *Sphaerochaeta coccoides* *Vibrio harveyi*	↑Glycoside hydrolase ↑Glycan degradation ↑Pyrimidine metabolism ↑Chaperones and folding catalysts ↑Bacterial toxins ↑Glycosaminoglycan degradation ↑Glycosphingolipid biosynthesis ↑Lysosome ↑Protein digestion and absorption ↓Tropane, piperidine, pyridine alkaloid biosynthesis ↓Chitin and peptidoglycan cleaving ↓Antigen processing and presentation ↓Influenza A ↓MAPK signalling pathway ↓Endocytosis	
	Caecum (*n* = 8)	~Day 300 of age	α-diversity not measured β-diversity differences between high- and low-fatness pigs	*Bacteroides* *dorei* *Bacteroides fragilis* *Bacteroides helcogenes* *Bacteroides salanitronis* *Bacteroides thetaiotaomicron* *Megamonas hypermegale* *Megasphaera elsdenii* *Prevotella ruminicola* *Treponema succinifaciens*	*Escherichia coli* ***Eubacterium*** *rectale* *Oscillibacter valericigenes* *Parabacteroides distasonis* *Roseburia hominis* *Roseburia intestinalis*	↑Chaperones and folding catalysts ↑Glycosaminoglycan degradation ↑Glycosphingolipid biosynthesis ↑Glycoside hydrolase ↑Adipocytokine signalling ↑Cellular antigens ↑Protein digestion and absorption ↓Lipopolysaccharide biosynthesis ↓Cytoskeleton proteins ↓Two-component system ↓Transporters ↓Benzoate degradation ↓Flagellar assembly ↓Bacterial chemotaxis	

Taxa found to be similarly differentially abundant across the two studies are shown in bold; taxa for which opposing findings were observed are underlined. Only significant effects (*p* < 0.05) are shown. ^1^ Where only 16S rRNA gene sequencing was performed, functional capacity of the gut microbiome was inferred from the 16S rRNA gene sequence data using PICRUSt software, as noted. Where this is the case, we have omitted predicted pathways considered irrelevant for pigs, e.g., those relating to cancer, etc.

**Table 2:** Bacterial diversity within the gut microbiome of pigs ranked on feed efficiency and bacterial genera/species and related pathways/genes ^1^ linked with feed efficiency.

Trait (Methodology for Structural and Functional Analysis of Gut Microbiome)	Sample Type (Total Number of Pigs)	Age of Pigs	α- and β-Diversity Effects in More Feed-Efficient Animals	Genera/Species More Abundant in More Feed-Efficient Animals	Genera/Species Less Abundant in More Feed-Efficient Animals	Functional Analysis (↓ = Bacterial Pathways Less Abundant in More Feed-Efficient Animals; ↑ = Pathways More Abundant in More Feed-Efficient Animals) ^1^	Ref
Residual feed intake (16S rRNA gene sequencing (V3-V4) on Illumina MiSeq + PICRUSt predictions)	Faeces (*n* = 32 at each time point)	Weaning (~Day 27 of age)	No effect	***Bacteroides***		No effect	[23]
		D42 pw ^2^			***Streptococcus***	No effect	
		Day 138 pw		^3^ UN_***Christensenellaceae** Cellulosilyticum* **UN_*vadinBB60*** ***Bacteroides***	*Clostridium sensu stricto 1* *****Can. Saccharimonas*****	↑RE ^4^ processing ↓Biosynthesis of 2° metabolites	
	Caecum (*n* = 30)	Day 139 pw	No effect	***Actinobacillus** porcinus*	*Solobacterium*	↓Thiamine metabolism	
	Ileum (*n* = 24)	Day 139 pw	No effect	***Oscillibacter*** ***Methanobrevibacter*** ***Treponema berlinense***	*Rhodococcus* *Methanosphaera* *Clostridiales*	↑Bacterial invasion ↑Biosynthesis of amino acids (phenylalanine, tyrosine, tryptophan, valine, leucine, isoleucine) ↑Metabolism of C5-branched dibasic acid, terpenoids, polyketides ↑RE processing ↓PTS ^5^	
Residual feed intake (16S rRNA gene sequencing (V3-V4) on Illumina MiSeq + PICRUSt predictions) (3 different geographic locations (AT ^7^, NI ^8^, ROI ^9^, with 2 batches in ROI (ROI1, ROI2))	Faeces (*n* = 100)	Day 70 of age	No effect	UN_*S24.7* (AT) *Mucispirillum* (NI, AT)	UN_*S24.7* (NI)	No common effects	[24]
		Day 134 of age	No effect	***Roseburia*** (NI) ***Treponema** brennaborense* (AT) ***Methanobrevibacter*** (ROI1 and 2) UN_*BS11.gut.group* (ROI2) UN_*RF16* (ROI1, AT) ***Alistipes*** (ROI2) *Sedimentibacter* (ROI2) *UN_Family XIII* (ROI2) *Oribacterium* (NI) *Pseudobutyrivibrio* (NI) *Papillibacter* (ROI2) ^10^ UNID_***vadinBB60*** (ROI2) UN_*RF12 gut group* (ROI1) UN_*WCHB1.25* (ROI1) *Leeia* (ROI1) UN_*Succinivibrionaceae* (ROI) UN_*TA18* (ROI2) *Desulfovibrio* (AT) UN_*RF9* (AT) ***Akkermansia*** (ROI1)	*Mogibacterium* (NI) *Ruminococcaceae* IS ^6^ (ROI1) UN_*Ruminococcaceae* (NI) UN_*Erysipelotrichaceae* (NI) *Alloprevotella* (ROI2) UN_*Bacteroidetes* (ROI1) ***Can. Saccharimonas*** (ROI2) UN_*Defluviitaleaceae* (ROI1) *Lachnospira* (ROI1) *Marvinbryantia* (ROI2) *Oribacterium* (ROI1) ***Faecalibacterium***(ROI2) *Ruminococcus*(NI) *Subdoligranulum* (ROI2) *Asteroleplasma* (ROI2) *Erysipelotrichaceae* IS (ROI1) *Allisonella* (ROI1) *Mitsuokella* (ROI1) *Selenomonas* (ROI2) UN_*Veillonellaceae* (ROI2)	↑Biosynthesis of unsaturated fatty acids (only common results shown, i.e., these followed the same trend in both ROI batches)	
	Ileum (*n* = 76) (NI pigs not sampled)	Day 134 of age	Higher α-diversity (Shannon and Simpson indices) (ROI2) No effect for β-diversity	*Dietzia* (ROI2) *Leucobacter* (ROI2) *Rothia* (ROI2) *Saccharopolyspora* (ROI2) UN_*S24.7* (ROI2) UN_*Gastranaerophilales* (ROI2) *Oceanobacillus* (ROI2) *Paucisalibacillus* (ROI2) *Dolosicoccus* (ROI2) *Globicatella* (ROI2) *Enterococcus* (ROI2) ***Clostridium** sensu stricto 1*(ROI1) ***Blautia*** (ROI2) *Pseudobutyrivibrio* (ROI2) *Subdoligranulum* (ROI2) UN_***vadinBB60*** (ROI2) *Turicibacter* (ROI1) *Solobacterium* (ROI2) *Dialister* (ROI2) *Mitsuokella*(ROI2) *p1088 a5 gut group* (ROI2) *Desulfovibrio* (ROI2) ***Actinobacillus*** (ROI2) *Spirochaeta* (ROI2)	UN_*vadinBB60* (ROI1) *Helicobacter* (ROI1)	No common effects	
	Caecum (*n* = 76) (NI pigs not sampled)	Day 134 of age	Higher α-diversity (Shannon and Simpson indices) (ROI2) No effect for β-diversity	*Microbacterium* (ROI2) UN_*Coriobacteriaceae* (ROI2) ***Paludibacter*** (ROI2) *UN_RF16* (ROI1, ROI2) *dgA 11 gut group* (ROI2) *RC9 gut group* (ROI2) UN_*4c0d* (AT) UN_*Gastranaerophilales* (ROI2) UN_***Erysipelotrichaceae*** (ROI2) *Asteroleplasma* (ROI2) UN_*Family XIII* (ROI2) *Peptococcus* (ROI2) *Ruminococcaceae* IS (ROI2) *Papillibacter* (ROI2) UN_***vadinBB60*** (ROI2) UN_*RF12 gut group* (ROI2) *Sutterella* (ROI1) *Noviherbaspirillum* (ROI2) UN_*GR WP33 58* (ROI2) *Helicobacter* (ROI2) *Ruminobacter* (ROI1)	*Butyrivibrio* (ROI2) *Oscillospira* (ROI1) ***Streptococcus*** (ROI2) UN_*Veillonellaceae* (ROI1)	↑Inositol phosphate metabolism ↓Porphyrin/chlorophyll metabolism (only common results shown, i.e., these followed the same trend in both ROI batches)	
Feed conversion ratio ((16S rRNA gene sequencing (V4–V5) on Illumina HiSeq+ PICRUSt predictions))	Ileum (*n* = 24)	Day 141–142 of age	No effect	***Streptococcus*** *Ruminococcaceae* spp. ***Christensenellaceae** R7*	*Roseburia* *Prevotellaceae* spp. *Rikenellaceae RC9*	↓Infectious diseases	[25]
	Caecum (*n* = 24)	Day 141–142 of age	Higher α-diversity (Shannon index)	***Eubacterium**hallii* ***Clostridium** sensu stricto1* ***Lachnospiraceae*** sp. *Dialister* *Rikenellaceae RC9* *Prevotellaceae* sp. *Ruminiclostridium* ***Treponema*** ***Ruminococcus*** *p-1088-a5 gut group*	***Bacteroides*** *Parabacteroides* ***Eubacterium coprostanoligenes*** *Sutterella* *Lachnoclostridium*	↑Carbohydrate metabolism ↑Lipid metabolism	
	Colon (*n* = 24)	Day 141–142 of age	Higher α-diversity (Shannon and Chao1 indices) No effect for β-diversity	*Ruminococcaceae* spp. ***Ruminococcus*** ***Prevotella*** *Rikenellaceae RC9* *Anaerotruncus* ***Eubacterium coprostanoligenes*** *Lawsonia* *Dielma* ***Treponema*** *Hydrogenoanaerobacterium* *Ruminiclostridium*	***Bacteroides*** ***Streptococcus*** *Lachnospiraceae* sp. *Macrococcus* *Ruminiclostridium* ***Streptococcus*** *Prevotellaceae* spp.	↑Metabolism of amino acids ↑Signalling molecules and interaction ↑Metabolism of cofactors and vitamins ↑Digestive system ↑Glycan biosynthesis and metabolism ↑Folding, sorting, degradation ↑Immune system ↑Metabolism of terpenoids and polyketides	
Residual feed intake 2 farms ((16S rRNA gene sequencing (V3–V4) on Illumina MiSeq + PICRUSt predictions))	Colon (*n* = 40)	Day 105–141 of age	No effect	***Ruminococcus** flavefaciens*	*Collinsella*	No effect	[26]
Feed conversion ratio (Metagenomic shotgun sequencing on Illumina platform)	Caecum (*n* = 6 pigs from [22])	Day 141–142 of age	β-diversity differences No effect for α-diversity	***Lactobacillus** johnsonii* *Acetatifactor muris* *Firmicutes bacterium CAG:110 Firmicutes bacterium CAG:124* *Firmicutes bacterium CAG:475* ***Treponema** bryantii* ***Treponema berlinense*** ***Treponema** succinifaciens* ***Treponema** socranskii* ***Treponema** saccharophilum* ***Treponema** brennaborense* ***Butyricicoccus**_porcorum* *Clostridiales_bacterium* *Bacteroidales WCE2004* *Methanogenic archaeon* ***Clostridia** bacterium* ***Alistipes*** sp. ***Eubacterium** sp. CAG:180* *Verrucomicrobia* *Faecalibacterium* sp. ***Bacteroides sp**.* ***Clostridium*** *Fibrobacter* ***Ruminococcus*** ***Roseburia*** Butyrivibrio *Desulfurispirillum* ***Paludibacter*** ***Blautia*** ***Methanobrevibacter***	***Prevotella*** sp. ***Bacteroides*** sp. *Escherichia coli* ***Prevotella** stercorea* ***Prevotella** bryantii* ***Prevotella** ruminicola* ***Prevotella** timonensis* *Succinivibrio dextrinosolvens* *Succinatimonas* sp. *Clostridium* sp.	↑Metabolism of protein, nucleotide, cofactors, vitamins ↑Monosaccharide and energy transportation ↑13 Antibiotic resistance genes ↓12 Antibiotic resistance genes ↓Genetic information processing ↓Amino sugar and nucleotide sugar metabolism ↓Transport system	[27]
Feed conversion ratio (Metagenomic shotgun sequencing on Illumina HiSeq 2500)	Caecum (*n* = 8)	Day 166 of age	β-diversity differences α-diversity not measured	*Acetivibrio ethanolgignens* ***Lactobacillus** ruminis* ***Lactobacillus** amylophilus **Lactobacillus*** sp. ***Butyricicoccus*** sp. *UN_**Butyricicoccus*** sp. ***Clostridium** bolteae* ***Ruminococcus*** sp. ***Clostridium** clostridioforme* ***Clostridium** saccharolyticum* ***Christensenella*** sp. ***Erysipelotrichaceae*** sp. ***Roseburia** intestinalis* *Coprococcus* spp. ***Lachnospiraceae*** sp. ***Roseburia** hominis* ***Eubacterium** siraeum* *Butyrate producing bacterium*	***Prevotella***	↑ABC transporters ↑Bacterial chemotaxis ↑D-arginine and D-ornithine metabolism ↑Flagellar assembly ↑Lysine degradation ↑Phenylalanine metabolism ↑Sulphur relay system ↑Synthesis and degradation of ketone bodies ↑Two-component system ↓Nitrogen metabolism ↓Glycan degradation	[28] ^11^
Residual feed intake (16S rRNA gene sequencing (V4) on Illumina MiSeq and metagenomic shotgun sequencing on Illumina HiSeq 2500)	Faeces (*n* = 18)	Day 140 of age	Not determined	*Aequorivita sublithincola* *Ammonifex degensii* ***Akkermansia*** *Bacteroidales bacterium CF* *Brachyspira pilosicoli* *Butyrate-producing bacterium* ***Clostridium** cellulosi* ***Clostridium** clariflavum* *Coriobacterium glomerans* *Desulfatibacillum alkenivorans* *Desulfovibrio desulfuricans* *Desulfovibrio vulgaris* *Eggerthella lenta* *Ethanoligenens harbinense* *Heliobacterium modesticaldum* ***Lactobacillus** casei* *Methylotenera versatilis* ***methao** valericigenes* *Paenibacillus graminis* *Paenibacillus sabinae* *Paenibacillus* sp. *Paenibacillus stellifer* *Pelobacter propionicus* *Podospora anserina* *Propionibacterium propionicum* *Pseudomonas fluorescens* *Pseudomonas mendocina* *Rikenellaceae bacterium M3* ***Streptococcus*** *gordonii* *Symbiobacterium thermophilum* *Syntrophobotulus glycolicus* *Tepidanaerobacter acetatoxydans* *Thermacetogenium phaeum* ***Treponema** denticola*	*Bordetella avium* ***Prevotella*** ***Faecalibacterium***	↑Nitrogen metabolism ↑Amino acid metabolism ↑Transport system ↑PPAR signalling pathway ↑Amino acid metabolism ↑Fatty acid metabolism ↑Adipocytokine signalling pathway ↓Metabolism and transport of monosaccharide ↓Signal transduction ↓Bacterial PTS ↓Phosphonate and phosphinate metabolism	[32]

Taxa found to be similarly differentially abundant across different studies are shown in bold; taxa for which opposing findings were observed are underlined. Only significant effects (*p* < 0.05) are shown. In some cases, operational taxonomic unit (OTU) detail has been removed. ^1^ Where only 16S rRNA gene sequencing was performed, functional capacity of the gut microbiome was inferred from the 16S rRNA gene sequence data using PICRUSt software, as noted. Where this is the case, we have omitted predicted pathways considered irrelevant for pigs, e.g., those relating to cancer, etc. ^2^ pw = post-weaning; ^3^ UN_ = uncultured (Note: Uncultured phyla have been omitted); ^4^ RE = restriction enzyme; ^5^ PTS = phosphotransferase system; ^6^ IS = *Incertae sedis*; ^7^ AT = Austria; ^8^ NI = Northern Ireland; ^9^ ROI = Republic of Ireland; ^10^ UNID_ = unidentified. ^11^ Only top 20 differentially enriched species listed as in the publication.

## References

[1] Cullen J., Lawlor P.G., Gardiner G.E., Bailey M., Stokes C. (2020). Microbiological services delivered by the pig gut microbiome. Understanding Gut Microbiomes as Targets for Improving Pig Gut Health.

[2] Deusch S., Tilocca B., Camarinha-Silva A., Seifert J. (2015). News in livestock research-use of Omics-technologies to study the microbiota in the gastrointestinal tract of farm animals. Comp. Struct. Biotechnol. J..

[3] Tröscher-Mußotter J., Tilocca B., Stefanski V., Seifert J. (2019). Analysis of the bacterial and host proteins along and across the porcine gastrointestinal tract. Proteomes.

[4] Looft T., Allen H.K., Cantarel B.L., Levine U.Y., Bayles D.O., Alt D.P., Henrissat B., Stanton T.B. (2014). Bacteria, phages and pigs: The effects of in-feed antibiotics on the microbiome at different gut locations. ISME J..

[5] Han G.G., Lee J.Y., Jin G.D., Park J., Choi Y.H., Chae B.J., Kim E.B., Choi Y.J. (2017). Evaluating the association between body weight and the intestinal microbiota of weaned piglets via 16S rRNA sequencing. Appl. Microbiol. Biotechnol..

[6] Yang H., Huang X., Fang S., Xin W., Huang L., Chen C. (2016). Uncovering the composition of microbial community structure and metagenomics among three gut locations in pigs with distinct fatness. Sci. Rep..

[7] Lu D., Tiezzi F., Schillebeeckx C., McNulty N.P., Schwab C., Shull C., Maltecca C. (2018). Host contributes to longitudinal diversity of fecal microbiota in swine selected for lean growth. Microbiome.

[8] Lau S.K.P., Woo P.C.Y., Woo G.K.S., Fung A.M.Y., Ngan A.H.Y., Song Y., Liu C., Summanen P., Finegold S.M., Yuen K. (2006). Bacteraemia caused by *Anaerotruncus colihominis* and emended description of the species. J. Clin. Pathol..

[9] Yekani M., Baghi H.B., Naghili B., Vahed S.Z., Sóki J., Memar M.Y. (2020). To resist and persist: Important factors in the pathogenesis of *Bacteroides fragilis*. Microb. Pathog..

[10] Togo A.H., Diop A., Dubourg G., Khelaifia S., Richez M., Armstrong N., Maraninchi M., Fournier P.E., Raoult D., Million M. (2019). *Anaerotruncusmassiliensis* sp. nov., a succinate-producing bacterium isolated from human stool from an obese patient after bariatric surgery. New Microbes New Infect..

[11] Zhong X., Harrington J.M., Millar S.R., Perry I.J., O’Toole P.W., Phillips C.M. (2020). Gut microbiota associations with metabolic health and obesity status in older adults. Nutrients.

[12] Torres-Pitarch A., Gardiner G.E., Cormican P., Rea M., Crispie F., O’Doherty J.V., Cozannet P., Ryan T., Lawlor P.G. (2020). Effect of cereal soaking and carbohydrase supplementation on growth, nutrient digestibility and intestinal microbiota in liquid-fed grow-finishing pigs. Sci. Rep..

[13] Mach N., Berri M., Estellé J., Levenez F., Lemonnier G., Denis C., Leplat J.J., Chevaleyre C., Billon Y., Doré J. (2015). Early-life establishment of the swine gut microbiome and impact on host phenotypes. Environ. Microbiol. Rep..

[14] Singh R.P. (2019). Glycan utilisation system in Bacteroides and Bifidobacteria and their roles in gut stability and health. Appl. Microbiol. Biotechnol..

[15] Torres-Pitarch A., Gardiner G.E., Cormican P., Rea M., Crispie F., O’Doherty J.V., Cozannet P., Ryan T., Cullen J., Lawlor P.G. (2020). Effect of cereal fermentation and carbohydrase supplementation on growth, nutrient digestibility and intestinal microbiota in liquid-fed grow-finishing pigs. Sci. Rep..

[16] Holman D.B., Brunelle B.W., Trachsel J., Allena H.K. (2017). Meta-analysis to define a core microbiota in the swine gut. mSystems.

[17] Ramayo-Caldas Y., Mach N., Lepage P., Levenez F., Denis C., Lemonnier G., Leplat J.J., Billon Y., Berri M., Doré J. (2016). Phylogenetic network analysis applied to pig gut microbiota identifies an ecosystem structure linked with growth traits. ISME J..

[18] Bergamaschi M., Maltecca C., Schillebeeckx C., McNulty N.P., Schwab C., Shull C., Fix J., Tiezzi F. (2020). Heritability and genome-wide association of swine gut microbiome features with growth and fatness parameters. Sci. Rep..

[19] Fang S., Xiong X., Su Y., Huang L., Chen C. (2017). 16S rRNA gene-based association study identified microbial taxa associated with pork intramuscular fat content in feces and cecum lumen. BMC Microbiol..

[20] Bergamaschi M., Tiezzi F., Howard J., Huang Y.J., Gray K.A., Schillebeeckx C., McNulty N.P., Maltecca C. (2020). Gut microbiome composition differences among breeds impact feed efficiency in swine. Microbiome.

[21] Louis P., Scott K.P., Duncan S.H., Flint H.J. (2007). Understanding the effects of diet on bacterial metabolism in the large intestine. J. Appl. Microbiol..

[22] Maltecca C., Lu D., Schillebeeckx C., McNulty N.P., Schwab C., Shull C., Tiezzi F. (2019). Predicting growth and carcass traits in swine using microbiome data and machine learning algorithms. Sci. Rep..

[23] McCormack U.M., Curião T., Buzoianu S.G., Prieto M.L., Ryan T., Varley P., Crispie F., Magowan E., Metzler-Zebeli B.U., Berry D. (2017). Exploring a possible link between the intestinal microbiota and feed efficiency in pigs. Appl. Environ. Microbiol..

[24] McCormack U.M., Curião T., Metzler-Zebeli B.U., Wilkinson T., Magowan E., Berry D.P., Reyer H., Prieto M.L., Buzoianu S.G., Harrison M. (2019). Porcine feed efficiency-associated intestinal microbiota and physiological traits: Finding consistent cross-locational biomarkers for residual feed intake. mSystems.

[25] Quan J., Cai G., Ye J., Yang M., Ding R., Wang X., Zheng E., Fu D., Li S., Zhou S. (2018). A global comparison of the microbiome compositions of three gut locations in commercial pigs with extreme feed conversion ratios. Sci. Rep..

[26] Vigors S., O’ Doherty J.V., Sweeney T. (2020). Colonic microbiome profiles for improved feed efficiency can be identified despite major effects of farm of origin and contemporary group in pigs. Animal.

[27] Quan J., Wu Z., Ye Y., Peng L., Wu J., Ruan D., Qiu Y., Ding R., Wang X., Zheng E. (2020). Metagenomic characterization of intestinal regions in pigs with contrasting feed efficiency. Front. Microbiol..

[28] Tan Z., Yang T., Wang Y., Xing K., Zhang F., Zhao X., Ao F., Chen S., Liu J., Wang C. (2017). Metagenomic analysis of cecal microbiome identified microbiota and functional capacities associated with feed efficiency in landrace finishing pigs. Front. Microbiol..

[29] Waters J.L., Ley R.E. (2019). The human gut bacteria *Christensenellaceae* are widespread, heritable, and associated with health. BMC Biol..

[30] Niu Q., Li P., Hao S., Zhang Y., Kim S.W., Li H., Ma X., Gao S., He L., Wu W. (2015). Dynamic distribution of the gut microbiota and the relationship with apparent crude fiber digestibility and growth stages in pigs. Sci. Rep..

[31] Rosewarne C.P., Cheung J.L., Smith W.J.M., Evans P.N., Tomkins N.W., Denman S.E., Cuív P.Ó., Morrison M. (2012). Draft genome sequence of *Treponema* sp. strain JC4, a novel spirochete isolated from the bovine rumen. J. Bacteriol..

[32] Yang H., Huang X., Fang S., He M., Zhao Y., Wu Z., Yang M., Zhang Z., Chen C., Huang L. (2017). Unravelling the fecal microbiota and metagenomic functional capacity associated with feed efficiency in pigs. Front. Microbiol..

[33] Million M., Angelakis E., Maraninchi M., Henry M., Giorgi R., Valero R., Vialettes B., Raoult D. (2013). Correlation between body mass index and gut concentrations of *Lactobacillus reuteri*, *Bifidobacterium animalis*, *Methanobrevibacter smithii* and *Escherichia coli*. Int. J. Obes..

[34] Nørskov-Lauritsen N. (2014). Classification, identification, and clinical significance of *Haemophilus* and *Aggregatibacter* species with host specificity for humans. Clin. Microbiol. Rev..

[35] Li J., Sung C.Y.J., Lee N., Ni Y., Pihlajamäki J., Panagiotou G., El-Nezami H. (2016). Probiotics modulated gut microbiota suppresses hepatocellular carcinoma growth in mice. Proc. Natl. Acad. Sci. USA.

[36] Cani P.D., de Vos W.M. (2017). Next-Generation Beneficial Microbes: The case of *Akkermansia muciniphila*. Front. Microbiol..

[37] Valeriano V.D.V., Balolong M.P., Kang D.K. (2016). Probiotic roles of *Lactobacillus* sp. in swine: Insights from gut microbiota. J. Appl. Microbiol..

[38] Vigors S., Sweeney T., O’Shea C.J., Kelly A.K., O’Doherty J.V. (2016). Pigs that are divergent in feed efficiency, differ in intestinal enzyme and nutrient transporter gene expression, nutrient digestibility and microbial activity. Animal.

[39] Songer G., Francisco A.U. (2005). Clostridial enteric infections in pigs. J. Vet. Diagn. Investig..

[40] Kaakoush N.O. (2015). Insights into the Role of *Erysipelotrichaceae* in the Human Host Front. Cell Infect Microbiol..

[41] Weishaar R., Wellmann R., Camarinha-Silva A., Rodehutscord M., Bennewitz J. (2020). Selecting the hologenome to breed for an improved feed efficiency in pigs-A novel selection index. J. Anim. Breed. Genet..

[42] Almeida D., Machado D., Andrade J.C., Mendo S., Gomes A.M., Freitas A.C. (2020). Evolving trends in next-generation probiotics: A 5W1H perspective. Crit. Rev. Food Sci. Nutr..

[43] Krzyściak W., Pluskwa K.K., Jurczak A., Kościelniak D. (2013). The pathogenicity of the *Streptococcus* genus. Eur. J. Clin. Microbiol. Infect. Dis..

[44] Pessione E. (2012). Lactic acid bacteria contribution to gut microbiota complexity: Lights and shadows. Front. Cell. Infect. Microbiol..

[45] Hols P., Ledesma-García L., Gabant P., Mignolet J. (2019). Mobilization of microbiota commensals and their bacteriocins for therapeutics. Trends Microbiol..

[46] Sommer F., Nookaew I., Sommer N., Fogelstrand P., Bäckhed F. (2015). Site-specific programming of the host epithelial transcriptome by the gut microbiota. Genome Biol..

[47] Grubbs J.K., Fritchen A.N., Huff-Lonergan E., Gabler N.K., Lonergan S.M. (2013). Selection for residual feed intake alters the mitochondria protein profile in pigs. J. Proteomics..

[48] Reyer H., Oster M., Magowan E., Muráni E., Sauerwein H., Dannenberger D., Kuhla B., Ponsuksili S., Wimmers K. (2018). Feed-efficient pigs exhibit molecular patterns allowing a timely circulation of hormones and nutrients. Physiol. Genomics.

[49] Vincent A., Louveau I., Gondret F., Tréfeu C., Gilbert H., Lefaucheur L. (2015). Divergent selection for residual feed intake affects the transcriptomic and proteomic profiles of pig skeletal muscle. J. Anim. Sci..

[50] Reyer H., Oster M., McCormack U.M., Muráni E., Gardiner G.E., Ponsuksili S., Lawlor P.G., Wimmers K. (2020). Host-microbiota interactions in ileum and caecum of pigs divergent in feed efficiency contribute to nutrient utilization. Microorganisms.

[51] Metzler-Zebeli B.U., Lawlor P.G., Magowan E., McCormack U.M., Curião T., Hollmann M., Ertl R., Aschenbach J.R., Zebeli Q. (2017). Finishing pigs that are divergent in feed efficiency show small differences in intestinal functionality and structure. PLoS ONE.

[52] Morrison D.J., Preston T. (2016). Formation of short chain fatty acids by the gut microbiota and their impact on human metabolism. Gut Microbes.

[53] Broom L., Kogut M. (2018). Gut immunity: Its development and reasons and opportunities for modulation in monogastric production animals. Anim. Health Res. Rev..

[54] McKenzie C., Tan J., Macia L., Mackay C.R. (2017). The nutrition-gut microbiome-physiology axis and allergic diseases. Immunol. Rev..

[55] Kaiko G.E., Stappenbeck T.S. (2014). Host-microbe interactions shaping the gastrointestinal environment. Trends Immunol..

[56] Zhao W., Wang Y., Liu S., Huang J., Zhai Z., He C., Ding J., Wang J., Wang H., Fan W. (2015). The dynamic distribution of porcine microbiota across different ages and gastrointestinal tract segments. PLoS ONE.

[57] Mauch E.D., Young J.M., Serão N.V.L., Hsu W.L., Patience J.F., Kerr B.J., Weber T.E., Gabler N.K., Dekkers J.C.M. (2018). Effect of lower-energy, higher-fiber diets on pigs divergently selected for residual feed intake when fed higher-energy, lower-fiber diets. J. Anim. Sci..

[58] Barea R., Dubois S., Gilbert H., Sellier P., van Milgen J., Noblet J. (2010). Energy utilization in pigs selected for high and low residual feed intake. J. Anim. Sci..

[59] Montagne L., Loisel F., Le Naou T., Gondret F., Gilbert H., Le Gall M. (2014). Difference in short-term responses to a high-fiber diet in pigs divergently selected for residual feed intake. J. Anim. Sci..

[60] Harris A.J., Patience J.F., Lonergan S.M., Dekkers J.C.M., Gabler N.K. (2012). Improved nutrient digestibility and retention partially explains feed efficiency gains in pigs selected for low residual feed intake. J. Anim. Sci..

[61] Vigors S., O’Doherty J.V., Kelly A.K., O’Shea C.J., Sweeney T. (2016). The effect of divergence in feed efficiency on the intestinal microbiota and the intestinal immune response in both unchallenged and lipopolysaccharide challenged ileal and colonic explants. PLoS ONE.

[62] Metzler-Zebeli B.U., Lawlor P.G., Magowan E., Zebeli Q. (2018). Interactions between metabolically active bacteria and host gene expression at the cecal mucosa in pigs of diverging feed efficiency. J. Anim. Sci..

[63] Li X., Shimizu Y., Kimura I. (2017). Gut microbial metabolite short-chain fatty acids and obesity. Biosci. Microbiota Food Health.

[64] Gondret F., Louveau I., Mourot J., Duclos M.J., Lagarrigue S., Gilbert H., van Milgen J. (2014). Dietary energy sources affect the partition of body lipids and the hierarchy of energy metabolic pathways in growing pigs differing in feed efficiency. J. Anim. Sci..

[65] Zaibi M.S., Stocker C.J., O’Dowd J., Davies A., Bellahcene M., Cawthorne M.A., Brown A.J., Smith D.M., Arch J.R. (2010). Roles of GPR41 and GPR43 in leptin secretory responses of murine adipocytes to short chain fatty acids. FEBS Lett..

[66] Samuel B.S., Shaito A., Motoike T., Rey F.E., Backhed F., Manchester J.K., Hammer R.E., Williams S.C., Crowley J., Yanagisawa M. (2008). Effects of the gut microbiota on host adiposity are modulated by the short-chain fatty-acid binding G protein-coupled receptor, Gpr41. Proc. Natl. Acad. Sci. USA.

[67] Jørgensen J.R., Clausen M.R., Mortensen P.B. (1997). Oxidation of short and medium chain C2-C8 fatty acids in Sprague -Dawley rat colonocytes. Gut.

[68] Shimizu H., Masujima Y., Ushiroda C., Mizushima R., Taira S., Ohue-Kitano R., Kimura I. (2019). Dietary short-chain fatty acid intake improves the hepatic metabolic condition via FFAR3. Sci. Rep..

[69] Saika A., Nagatake T., Kunisawa J. (2019). Host- and microbe-dependent dietary lipid metabolism in the control of allergy, inflammation, and immunity. Front. Nutr..

[70] Lochmiller R.L., Deerenberg C. (2000). Trade-offs in evolutionary immunology: Just what is the cost of immunity?. Oikos.

[71] Rauw W.M. (2012). Immune response from a resource allocation perspective. Front. Genet..

[72] Frosali S., Pagliari D., Gambassi G., Landolfi R., Pandolfi F., Cianci R. (2015). How the intricate interaction among toll-like receptors, microbiota, and intestinal immunity can influence gastrointestinal pathology. J. Immunol. Res..

[73] Mani V., Harris A.J., Keating A.F., Weber T.E., Dekkers J.C.M., Gabler N.K. (2013). Intestinal integrity, endotoxin transport and detoxification in pigs divergently selected for residual feed intake. J. Anim. Sci..

[74] Cooke C.L., An H.J., Kim J., Canfield D.R., Torres J., Lebrilla C.B., Solnick J.V. (2009). Modification of gastric mucin oligosaccharide expression in rhesus macaques after infection with *Helicobacter pylori*. Gastroenterology.

[75] Day C.J., Semchenko E.A., Korolik V. (2012). Glycoconjugates play a key role in *Campylobacter jejuni* infection: Interactions between host and pathogen. Front. Cell. Infect. Microbiol..

[76] Quintana-Hayashi M.P., Mahu M., De Pauw N., Boyen F., Pasmans F., Martel A., Premaratne P., Fernandez H.R., Teymournejad O., Vande Maele L. (2015). The levels of *Brachyspira hyodysenteriae* binding to porcine colonic mucins differ between individuals, and binding is increased to mucins from infected pigs with de novo MUC5AC synthesis. Infect. Immun..

[77] Sun M., Wu W., Liu Z., Cong Y. (2017). Microbiota metabolite short chain fatty acids, GPR, and inflammatory bowel diseases. J. Gastroenterol..

[78] Chang P.V., Hao L., Offermanns S., Medzhitov R. (2014). The microbial metabolite butyrate regulates intestinal macrophage function via histone deacetylase inhibition. Proc. Natl. Acad. Sci. USA.

[79] Nakanishi N., Tashiro K., Kuhara S., Hayashi T., Sugimoto N., and Tobe T. (2019). Regulation of virulence by butyrate sensing in enterohaemorrhagic *Escherichia coli*. Microbiology.

[80] Jacobson A., Lam L., Rajendram M., Tamburini F., Honeycutt J., Pham T., Van Treuren W., Pruss K., Stabler S.R., Lugo K. (2018). A gut commensal-produced metabolite mediates colonization resistance to *Salmonella* infection. Cell Host Microbe.

[81] De Ridder L., Maes D., Dewulf J., Pasmans F., Boyen F., Haesebrouck F., Méroc E., Butaye P., Van der Stede Y. (2013). Evaluation of three intervention strategies to reduce the transmission of *Salmonella* Typhimurium in pigs. Vet. J..

[82] Luethy P.M., Huynh S., Ribardo D.A., Winter S.E., Parker C.T., Hendrixson D.R. (2017). Microbiota-derived short-chain fatty acids modulate expression of *Campylobacter jejuni* determinants required for commensalism and virulence. mBio.

[83] Stanley D., Hughes R.J., Geier M.S., Moore R.J. (2016). Bacteria within the gastrointestinal tract microbiota correlated with improved growth and feed conversion: Challenges presented for the identification of performance enhancing probiotic bacteria. Front. Microbiol..

[84] Browne H.P., Forster S.C., Anonye B.O., Kumar N., Neville B.A., Stares M.D., Goulding D., Lawley T.D. (2016). Culturing of ‘unculturable’ human microbiota reveals novel taxa and extensive sporulation. Nature.

[85] Marsh A.J., Azcarate-Peril M.A. (2020). Hologenomics: The interaction between host, microbiome and diet. Reference Module in Food Science..

[86] Rychlik J.L., May T. (2000). The effect of a methanogen, *Methanobrevibacter smithii*, on the growth rate, organic acid production, and specific ATP activity of three predominant ruminal cellulolytic bacteria. Curr. Microbiol..

[87] Bakken J.S., Borody T., Brandt L.J., Brill J.V., Demarco D.C., Franzos M.A., Kelly C., Khoruts A., Louie T., Martinelli L.P. (2011). Fecal Microbiota Transplantation Workgroup. Treating *Clostridium difficile* infection with fecal microbiota transplantation. Clin. Gastroenterol. Hepatol..

[88] Canibe N., O’Dea M., Abraham S. (2019). Potential relevance of pig gut content transplantation for production and research. J. Anim. Sci. Biotechnol..

[89] McCormack U.M., Curião T., Wilkinson T., Metzler-Zebeli B.U., Reyer H., Ryan T., Calderon-Diaz J.A., Crispie F., Cotter P.D., Creevey C.J. (2018). Fecal microbiota transplantation in gestating sows and neonatal offspring alters lifetime intestinal microbiota and growth in offspring. mSystems.

[90] McCormack U.M., Curião T., Metzler-Zebeli B.U., Wilkinson T., Reyer H., Crispie F., Cotter P.D., Creevey C.J., Gardiner G.E., Lawlor P.G. (2019). Improvement of feed efficiency in pigs through microbial modulation via fecal microbiota transplantation in sows and dietary supplementation of inulin in offspring. Appl. Environ. Microbiol..

[91] Hu J., Ma L., Nie Y., Chen J., Zheng W., Wang X., Xie C., Zheng Z., Wang Z., Yang T. (2018). A microbiota-derived bacteriocin targets the host to confer diarrhea resistance in early-weaned piglets. Cell Host Microbe.

[92] Metzler-Zebeli B.U., Canibe N., Montagne L., Freire J., Bosi P., Prates J.A.M., Tanghe S., Trevisi P. (2017). Resistant starch reduces large intestinal pH and promotes fecal lactobacilli and bifidobacteria in pigs. Anim. Int. J. Anim. Biosci..

[93] Biagi G., Piva A., Moschini M., Vezzali E., Roth F.X. (2007). Performance, intestinal microflora, and wall morphology of weanling pigs fed sodium butyrate. J. Anim. Sci..

[94] Fang C.L., Sun H., Wu J., Niu H.H., Feng J. (2014). Effects of sodium butyrate on growth performance, haematological and immunological characteristics of weanling piglets. J. Anim. Physiol. Anim. Nutr..

[95] Sun W., Sun J., Li M., Xu Q., Zhang X., Tang Z., Chen J., Zhen J., Sun Z. (2020). The effects of dietary sodium butyrate supplementation on the growth performance, carcass traits and intestinal microbiota of growing-finishing pigs. J. Appl. Microbiol..

[96] Hirschberg S., Gisevius B., Duscha A., Haghikia A. (2019). Implications of diet and the gut microbiome in neuroinflammatory and neurodegenerative diseases. Int. J. Mol. Sci..

[97] Tilocca B., Burbach K., Heyer C.M.E., Hoelzle L.E., Mosenthin R., Stefanski V., Camarinha-Silva A., Seifert J. (2017). Dietary changes in nutritional studies shape the structural and functional composition of the pigs’ fecal microbiome-from days to weeks. Microbiome.

